# Scaling of internal joint distance in the elbow of small‐ to medium‐sized mammals: Implications for range of motion analyses

**DOI:** 10.1111/joa.70116

**Published:** 2026-02-06

**Authors:** Adrian Scheidt, Alina C. E. Renk, John A. Nyakatura

**Affiliations:** ^1^ AG Vergleichende Zoologie, Institut für Biologie Humboldt Universität Zu Berlin Berlin Germany

**Keywords:** allometry, APSE, body mass, cartilage thickness, ROM

## Abstract

Cartilage thickness in mammalian joints increases with higher body masses. Contradicting previous studies found this increase to be positive allometric or negative allometric. Since approaches like computational modelling of animal locomotion, muscle moment arms, and joint dynamics rely on estimates of joint spacing (JS), it is important to accurately estimate an animal's cartilage thickness based on body mass. Here, we measured ex vivo internal joint distances (IJDs) or bone‐to‐bone distances on CT scans of fresh cadaveric forelimbs in a sample of small‐ to medium‐sized mammals. IJDs were measured in the humero‐ulnar and humero‐radial joint. We find IJDs to scale isometrically in both joints across the entire sample, with positive allometric tendencies only within a subsample of cursorial species and only in the humero‐ulnar articulation. The previously reported positive allometry can be linked to a cursorial sampling bias, and negative allometry results from a size constraint, acting on larger mammals than sampled here. Additionally, the IJDs were not affected by limb poses (i.e., flexed to extended, supinated, pronated). In rats and guinea pigs of varying sizes, we observed intraspecific isometric scaling with slight positive trends. This suggests that theoretically greater absolute forces—resulting from increased body mass with similar posture—only marginally contribute to relatively thicker cartilage in small‐ to medium‐sized mammals. Further, we conducted a “range of motion” (ROM) analysis in the humero‐ulnar joint of rats, guinea pigs, and maras, thus species of increasing body mass and level of cursoriality. ROM was assessed with varying estimated JS. Differences in the results are most biologically reasonable when allowing all six degrees of freedom (DOF, three rotational and three translational). Mobility decreases with increasing body mass and level of cursoriality, facilitated by increased restriction of movement to a single axis of flexion and extension. Such a trend persisted regardless of whether JS thresholds were estimated using intraspecific or interspecific regression models. The results suggest that variations in elbow mobility are less influenced by applied JS than by the morphological characteristics of the bones forming the joint. This observation has implications for future comparative studies of mammalian elbow function. Still, more research is needed to separate body mass, degree of cursoriality, or locomotor type as factors for elbow mobility.

## Graphical abstract

We investigated the internal joint distances (IJDs) of the humero‐ulnar and humero‐radial joint, within a sample of 15 small‐ to medium‐sized mammals and report isometric results. We also found that joint poses had no effect on IJDs and that IJDs scale isometrically within small species. Last, we conducted a “Range of Motion” analysis in the humero‐ulnar joint applying various joint spacing (JS) estimates and found that joint morphology, body mass, or locomotor type have a stronger effect than modelled JS on elbow mobility.

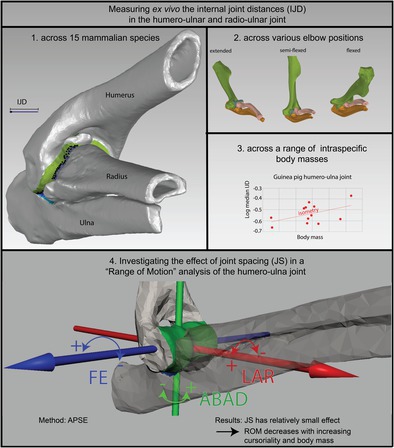

## 1 INTRODUCTION

The mammalian elbow joint usually comprises the humero‐ulnar, humero‐radial, and proximal radial‐ulnar articulations and is serving various functions. Flexion and extension of the humero‐ulnar joint serve mainly to provide stability and keep the posture upright during locomotion (Fischer & Blickhan, 2006; Fujiwara & Hutchinson, 2012; Hildebrand, 1974), while contributing little to forward progression (Fischer & Blickhan, 2006; Kuznetsov, 1985). Generally, the humero‐ulnar joint is regarded as a “single axis hinge joint,” limited to flexion and extension (FE) (Bonnan et al., 2016; Brocklehurst et al., 2025; Fujiwara, 2009; Hildebrand, 1974; Jenkins, 1973). Yet, recent studies indicate ulnar abduction/adduction (ABAD), and long axis rotation (LAR) is possible to some degree, based on ex vivo (Brocklehurst et al., 2025), in vivo (Scheidt et al., 2022), and in silico measures (Richards et al., 2021). Generally, the shape of the anatomical components of a joint, for example, bones’ articulation sites, epiphyses, and soft tissues like cartilage and ligaments, affects the function and mobility of a joint (Arnold et al., 2014; Demuth et al., 2025; Figueirido & Janis, 2011; Hildebrand, 1974; Manafzadeh, 2023).

The gap between the bones of any synovial joint in a mammalian limb (including the joints of the elbow) is composed of cartilage and synovial fluid (Hildebrand, 1974; Liao et al., 2019). The articular cartilage serves as a shock absorber to transmit forces and, together with the synovial fluid, enables low friction movement of the joint (Grodzinsky et al., 2000; Hildebrand, 1974; Malda et al., 2013; Marquez‐Florez et al., 2024). Absolute forces acting within the joints theoretically increase in terrestrial species with higher body mass (BM) (Bertram & Biewener, 1988; Biewener, 1983, 2005; Biewener & Patek, 2018; Simon, 1970). As cartilage is subjected to these forces, its measured thickness expectedly increases in the limb joints of terrestrial mammals as BM increases too (Bonnan et al., 2013; Frisbie et al., 2006; Malda et al., 2013; Simon, 1970; Stockwell, 1971). The cube‐square‐law stipulates that volume (and therefore BM) increases with the third power of a unit length, while the area of the supporting structures increases by the second power. To maintain safety factors and avoid injury due to the higher loadings (Biewener, 1989, 1990, 2005), mammals respond through behavioral and postural adjustments (Biewener, 2005; Biewener & Patek, 2018). Also, bone mass of supporting structures increases over‐proportionally, thus with positive allometry (Biewener & Patek, 2018; Christiansen, 2002; Doube et al., 2011; Scheidt et al., 2019). By contrast, cartilage response mechanisms to increased demands imposed by higher BMs are less well understood (Bonnan et al., 2013; Malda et al., 2013). Somewhat contradictorily, positive allometry in the cartilage thickness of the tibiofemoral (McLure et al., 2012), hip, ankle, shoulder, elbow (Simon, 1970), the knee joints, and underneath the patella across mammalian BMs have been reported (McLure et al., 2012; Simon, 1970; Stockwell, 1971). Yet, Malda et al. (2013) found negative allometry in cartilage thickness of femoral condyles across a larger sampled mammalian BM range. Previous research of cartilage thickness in (non‐human) mammals thus mostly focused on the knee joint (see Malda et al., 2013 and references therein). Additional comparative cartilage thickness measures are limited to small sample sizes of 5 to 7 species (Frisbie et al., 2006; Simon, 1970; Stockwell, 1971). This study presents original data on median internal joint distances at the elbow and its scaling relationship with BM for a comparative sample of small‐ to medium‐sized terrestrial mammals.

Because internal joint distances can be quantified directly from morphology, they provide a valuable link between anatomical variation and comparative studies of joint form and function (Demuth et al., 2023; Manafzadeh, 2023). When modelling a joint's theoretical “range of motion” (ROM), the estimated distance between articulating surfaces—hereafter called joint spacing (JS)—strongly affects the results (Arnold et al., 2014; Hutchinson et al., 2005; Jones, Brocklehurst, & Pierce, 2021; Lee et al., 2023; Manafzadeh & Gatesy, 2021; Wiseman et al., 2022). This necessitates accurate JS assessments. Estimating ROM has diverse applications in biomechanical and evolutionary studies (reviewed by Manafzadeh, 2023), such as modelling locomotion and movement in extant species (e.g., Lee et al., 2023; Manafzadeh & Padian, 2018; Merten et al., 2023; Regnault & Pierce, 2018) or calculating muscle moment arms (Brocklehurst et al., 2022; Löffler et al., 2022). In paleontological research, ROM is used to model locomotion (Bishop et al., 2021; Herbst, Manafzadeh, et al., 2022; Hutchinson et al., 2005; Molnar et al., 2021) and posture (Bishop & Pierce, 2024; Demuth et al., 2020; Fahn‐Lai et al., 2018; Jannel et al., 2019; Manafzadeh et al., 2024; Nyakatura et al., 2015; Pierce et al., 2012; Richards et al., 2021) in extinct taxa. Hence, in silico methods modelling locomotor biomechanics and joint function (e.g., Demuth et al., 2023; Falkingham, 2025; Manafzadeh et al., 2025) rely on accurate JS approximations (Arnold et al., 2014; Jones, Brocklehurst, et al., 2021; Lee et al., 2023; Manafzadeh & Gatesy, 2021; Nyakatura et al., 2019).

We define internal joint distance (IJD) as the shortest distance from one point on the bony articular surface to the articular surface of the other bone in the same joint. Concordantly, average IJD is coupled to average cartilage thickness, with computational modelling studies assuming the JS as twice the average cartilage thickness. In this study, we thus distinguish IJD (measured distances within joints) from JS (estimates used in computational models). Several methods have been proposed to estimate JS: empirical measurements at multiple sites within a joint using x‐ray or CT data (e.g., Arnold et al., 2014; Manafzadeh et al., 2021; Merten et al., 2023; Regnault et al., 2021; Richards et al., 2021), extrapolations from cartilage–body mass (BM) correlations (Holliday et al., 2010; Regnault et al., 2021; Richards et al., 2021; Simon, 1970; Stockwell, 1971), or geometric fits of primitives (e.g., spheres, cylinders) to articular surfaces (Brocklehurst et al., 2022; Demuth et al., 2020). Because JS estimation influences ROM results, sensitivity analyses with varying JS values are often included (e.g., Herbst, Eberhard, et al., 2022; Jannel et al., 2019; Merten et al., 2023; Richards et al., 2021). Given recent advances in accuracy for (paleo‐)biological inferences based on the analysis of ROM (Bishop et al., 2023; Manafzadeh et al., 2021, 2024, 2025), we regard the currently available methods for estimation of JS as insufficient. For example, the described methods for JS estimation fail to accurately capture the non‐uniform cartilage thickness, and therefore IJDs, within single joints (Malda et al., 2013; McLure et al., 2012). This is especially relevant for ROM analyses when ex vivo or in vivo bone articulation data (for example through CT scans) are not available, as is the case for the analysis of fossils. Also, the IJDs may vary according to certain limb poses (like flexed/extended, pronated/supinated). Therefore, it is tested here how limb poses affect the IJDs. Further, the unresolved scaling relationship between BM and average IJD complicates the inclusion of species into analyses for which no direct IJDs (to inform JS) can be obtained, even if BM estimates are available.

Besides accurate JS estimation, ROM analyses depend on how rotations and translations are permitted around a joint's center of rotation (COR). Each COR can be described by six degrees of freedom (DOF)—rotations around and translations along three axes (Bishop et al., 2023; Manafzadeh & Gatesy, 2021; Richards et al., 2021). Excluding translations (allowing only rotations) has the potential to miss rotational values present during in vivo locomotion and is thus regarded to be too restrictive. Allowing all six DOFs can be overly permissive, making interspecific comparisons difficult (Manafzadeh et al., 2024; Manafzadeh & Gatesy, 2021). Also, rotations and translations within the elbow joint are likely coupled (Regnault et al., 2021; Richards et al., 2021), thus omitting translations is biologically unrealistic. For analyses in hinge‐like joints, it has therefore been proposed to allow four DOFs—all rotations plus a single translation along the distraction–compression axis (Manafzadeh & Gatesy, 2021; Regnault et al., 2021; Regnault & Pierce, 2018). To identify biologically meaningful and comparable ROM protocols, we test multiple ROM setups varying in allowed DOFs and JS estimates.

Using complementary analytical approaches, our objectives are four‐fold: Interspecific scaling analysis (1). Establish the interspecific scaling relationship of median IJDs in the elbow joints for a comparative sample of small‐ to medium‐sized mammals. We measured the ex vivo IJDs in the humero‐ulnar and humero‐radial joints in 15 mammalian species, with published species mean BMs ranging from 1.2 g to 21,000 g (Smith et al., 2017; Wilson et al., 2016), covering mammals' most frequently occurring BMs (Brown et al., 1993; Lovegrove, 2001; Lovegrove & Haines, 2004). Pose spectrum analysis (2). Investigate the effect of limb poses on IJDs of the humero‐ulnar and humero‐radial joint. To achieve this, ex vivo IJDs were measured in multiple specimens of three rodent species with increasing degrees of cursoriality (rats, guinea pigs, mara. The cadavers were fixed in a spectrum of elbow poses (maximum flexion to extension, and maximum pronation and supination). Intraspecific scaling analysis (3). Investigate the intraspecific scaling relationship in IJDs to BM. Our sample of rats and guinea pigs consisting of individuals of varying sizes was again used to assess the intraspecific effect of body mass on median IJD. Range of motion analysis (4). Present a workflow and results in applying the scaling relationship of (1) and (3) to a ROM analysis of the humero‐ulnar joint of rats, guinea pigs, and maras to illustrate the significance of JS and DOFs allowed in comparative studies of form and function through computational modeling approaches.

## 2 MATERIALS AND METHODS

### 2.1 Interspecific scaling analysis (1)

20 specimens of 15 mammals were used in this study, with calculated BMs ranging from 1 g (Etruscan shrew) to 17,006 g (one of the three dog specimens, Table 1). This range includes the smallest mammal species, the Etruscan shrew, multiple common model organisms (e.g., mouse, rat), common pets (cat, dog), and wild animals (e.g., mole, banded mongoose). As mammals have a modal BM of just 100 g, the sample covers mammals' most frequently occurring BMs (Brown et al., 1993; Lovegrove, 2001; Lovegrove & Haines, 2004). The specimens were donated from various sources, and no animals were sacrificed for this study. All specimens used were adult with fused epiphyses (as visible in CT scans, see below). Epiphyses of rats were not fused, although they were adults and at least 10 weeks old (Dawson, 1925). Specimens had been stored in a freezer prior to this study. They were thawed and a single forelimb removed. The limbs were then fixated with wood and tight straps in a ca. 90° flexed elbow and pronated hand position. They were frozen again and subsequently scanned in a microfocus computed tomography scanner (μCT, XYLON FF20‐CT‐System, YXLON GmbH, Hamburg, Germany) with a resolution of 1.7 μm to 34.6 μm depending on limb size. Meshes of humerus, ulna, and radius were segmented in Dragonfly (Version 2024.1, Comet Technology Canada Inc., Montreal, Canada) and uploaded into Geomagic wrap (Version 2017.0.0, 3D Systems Inc., Rock Hill, SC, USA) (3D Systems, 2017). In our specimen‐based approach, the humeral circumference at the narrowest section (minimal humeral circumference, mHC) along the humeral diaphysis was measured of each specimen, to approximate its BM using Campione and Evans' (2012) regression data for mammals below 20 kg. It offers the advantage that the following methodology is universally applicable to future studies, where the BM of a specimen is unknown (as in paleobiological studies) and does not rely on rough species average data from the literature. Yet, we acknowledge that this sampling bias towards small‐ to medium‐sized mammals may limit applicability of the results for future studies involving much larger species. Multiple specimens of dog, cat, and mara were available, and the arithmetic mean of their results were used. As the estimated BM is less reliable in species with specialized humerus morphology, for example, fossorial species (Campione & Evans, 2012; Richards et al., 2019), we estimated the mHC of the mole based on literature BM ranges (Wilson et al., 2016) to include it in the regression analysis.

**TABLE 1:** Overview of the included species and estimated body masses (BM). Bold rows refer to mean values, calculated from multiple specimens listed in the rows above. Estimated BMs are calculated from the minimal humeral circumference (mHC) (Campione & Evans, 2012). The moles' estimated values used for the scaling analysis are reported in brackets (see text for details). Sources on BM ranges: Dogs (Smith et al., 2017), guinea pigs (Czarnecki & Adamski, 2016), cats (Hendriks et al., 1997), others (Wilson et al., 2016). Sources for categorization of locomotor types: Dog (Janis & Figueirido, 2014), cat (Wilson et al., 2016), guinea pig (Buxton & Peck, 1989), mara (Elissamburu & Vizcaíno, 2004; Rocha‐Barbosa & Casinos, 2011; Seckel & Janis, 2008), mongoose (Taylor, 1976), all others (Wilson et al., 2016).

Species	Common name	mHC (mm)	Estimated BM (g)	BM range by literature (g)	locomotor category
*Canis familiaris*	Dog	41.2	17,006		
*Canis familiaris*	Dog	35.6	11,327		
*Canis familiaris*	Dog	19.4	2097		
***Canis familiaris***	**Dog**	**32**	**10,143**	**2600–47,000**	**Cursorial**
*Cavia porcellus*	Guinea pig	13	695	700–1100	(Semi‐)Cursorial
*Dolichotis patagonum*	Patagonian mara	27.6	5581		
*Dolichotis patagonum*	Patagonian mara	28.7	6226		
***Dolichotis patagonum***	**Patagonian mara**	**28.2**	**5903**	**7000‐9000**	**Cursorial**
*Felis catus*	Cat	22.9	3349		
*Felis catus*	Cat	23	3385		
*Felis catus*	Cat	22.9	3340		
***Felis catus***	**Cat**	**22.9**	**3358**	**2400–5000**	**Cursorial**
*Lutra lutra*	Eurasian otter	29.4	6696	5000‐14,000	Non‐cursorial (aquatic)
*Micromys minutus*	Eurasain Harvest Mouse	2.6	8	4–11	Non‐cursorial (generalist)
*Monodelphis domestica*	Short‐tailed opossum	7	127	58–110	Non‐cursorial (generalist)
*Mungos mungo*	Banded Mongoose	18.5	1837	890–1880	(Semi‐)Cursorial
*Mus musculus*	House mouse	3.5	18	12–39	Non‐cursorial (generalist)
*Rattus norvegicus*	Brown rat	7.1	128	195–540	Non‐cursorial (generalist)
*Sapajus apella*	Tufted capuchin	20.9	2581	1300–4800	Non‐cursorial (arboreal)
*Sciurus vulgaris*	Red squirrel	11.3	467	235–408	Non‐cursorial (arboreal)
*Suncus etruscus*	Etruscan shrew	1.2	1	1.2–2.7	Non‐cursorial (generalist)
*Talpa europaea*	European mole	12.7 (5.8)	645 (73)	72–128	Non‐cursorial (fossorial)
*Tamiops swinhoei*	Swinhoe's striped squirrel	6	82	88	Non‐cursorial (generalist)

**FIGURE 1:**
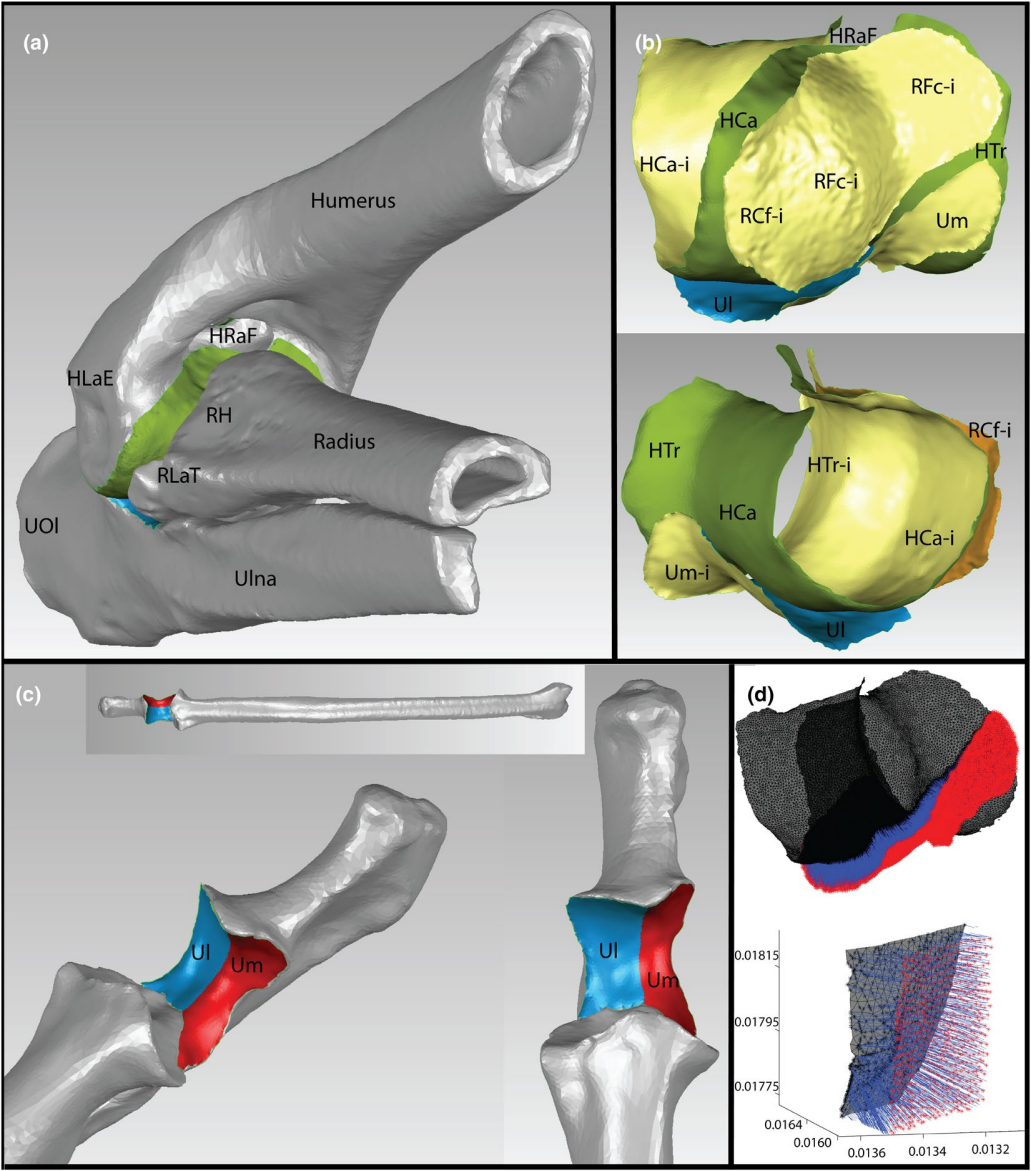
Workflow of the internal joint distance (IJD) analyses (1, 2, 3). (a) Bone meshes of the right elbow of a mara in distal cranio‐lateral view. Articulation surfaces of the humerus (green) and ulna (blue) are highlighted. (b) Extracted meshes of the articulation sites of a mara's right elbow with humeral trochlea in green, ulnar notch in blue, and radial head in orange. Note that the interior side (i) of the faces of the mesh are yellow. b top: Articulation sites in same view as a. b bottom: Articulation meshes in latero‐caudal view. (c) Right radioulna of mara, with the medial (red) and lateral (blue) part of the ulnar articulation site colored. Images are in cranial (top), medio‐distal (left), and disto‐cranial (right) view. (d) top: Exemplary screenshot of measured distances within the right humero‐ulnar joint of mara in roughly the same view as b top (cranio‐lateral view). The faces of the mesh of the humeral articulation site are black, the vertices of the ulnar articulation mesh are red, and the shortest connection from each ulnar vertex to the humeral articulation site is a blue line. Each blue line thus represents one measured IJD (see d bottom for details). The radial head is not shown here. (d) bottom: Magnified portion of a small section of d top, with the coordinate system indicating space in mm. Black asterixis represent the position of the shortest distance on the mesh of the humeral articulation site. Red asterixis are vertices of the mesh of the ulnar notch. HLaE, humeral lateral epicondyle; RH, radial head; RLaT, radial lateral tuberosity; UOl, ulnar olecranon; HCa, humeral capitulum; HRaF, humeral radial fossa; R, radial articulation site; RCf, radial capitular fovea; RFc, radial fovea capitis; Ul, ulnar lateral articulation site (inside ulnar notch); Um, ulnar medial articulation site (inside ulnar notch); −i, Interior side of mesh.

In Geomagic wrap, the articulation surfaces were selected either manually by the “Lasso Tool” or using the “Curves>Extract Tool,” depending on the shape of the surface. Selection was made along the edge of the articulation surface, deleting the rest of the bone (Figure 1a,b). The selected articulation site was re‐meshed to ensure uniform tessellation (“Remesh Tool”) of ca. 20,000 to 30,000 faces. Then, the meshes of the articulation sites were exported as .obj files.

Actual distances within the joints were calculated in Matlab using the function “point2trimesh()” (Frisch, 2025). It returns the shortest straight line from any query point to the nearest vertex, point on an edge, or face's surface of a triangulated mesh. It requires the vertices of one mesh (thus the query points), and the vertices and face coordinates of the other mesh imported as numeric matrices into Matlab. We chose measuring from distal points towards proximal faces as we assume that, under articulation, the whole surface (and thus vertices in the mesh files) of the smaller articulation site have a high percentage congruence to the larger articulation side. Additionally, this direction is also implemented in the ROM method applied here later. Note that 20,000–30,000 faces result in roughly 15,000 to 20,000 vertices. Any average IJD reported here is thus measured and averaged on 15,000–20,000 evenly spaced instances across the distal articulation site (Figure 1d). Preliminary results showed a plateau of IJDs, with the majority of faces having approximately similar distances (Figure S1). Given the plateau, we opted to base the regression analysis on median IJD per species.

To find differences in medial and lateral IJDs in the humero‐ulnar joint, the extracted .obj of the ulnar articulation site (trochlear notch) was split in two meshes along the central ridge of the trochlear notch, from the anconeal process to the coronoid process (Janis & Martín‐Serra, 2020), forming two new .obj files (Figure 1c). Then, the distance measures were repeated for each file (i.e., each part of the trochlear notch) separately.

With the mammalian skeleton generally, and the median IJD (*y*) scaling proportionally (*y* = *ax* ^*b*^) to BM (*x*), the data were log transformed (base 10). This resulted in log (*y*) = *b* log(*x*) + log(*a*), with a slope *b* < 1 referring to negative allometry, *b* = 1 to isometry, and *b* > 1 to positive allometry (Biewener & Patek, 2018; Malda et al., 2013; Scheidt et al., 2019; Warner et al., 2013). The resulting slopes (log mHC vs. log radial distances; log mHC vs. log ulnar distances; log mHC vs. log ulna lateral distances; log mHC vs. log ulna medial distances) were checked for a phylogenetic signal using a single, fully resolved, time‐calibrated phylogenetic tree of the study taxa, generated using the TimeTree database (Kumar et al., 2022). The R packages “readxl” (v 1.4.3; Wickham & Bryan, 2015), “ape” (v 5.8.1; Paradis & Schliep, 2019), and “phytools” (v 2.4.4; Revell, 2024) were used to estimate the parameter Blomberg's K (Blomberg et al., 2003) on the residuals. We chose Blomberg's K due to the relatively small sample size (*N* = 15, Münkemüller et al., 2012). The null hypothesis of no phylogenetic signal assuming Brownian motion when accounting for a specimen's body mass was tested for significance with α = 0.05. Additionally, we calculated the 95% confidence interval (CI) of the slope coefficient using the packages “dplyr” (v 1.1.4; Wickham et al., 2014) and “broom” (v 1.0.5; Robinson et al., 2014). If the CI included a value of 1.0, the slope was considered to indicate isometry.

As we noted a sampling bias in previous studies (Simon, 1970; Stockwell, 1971) towards cursorial species, and since the *ROM analysis* (4) focuses on a gradient in cursoriality, we assigned locomotor categories to our sample of taxa (Table 1). We then assessed if the scaling relationships between the “cursorial” and “other” locomotor types differed statistically. “Semi‐cursorial” and “cursorial” species were both considered “cursorial.” We fitted multiple linear regression models with an interaction term between them. The significance of the interaction term was evaluated using a *t*‐test on the corresponding regression coefficient, testing the null hypothesis that the slopes do not differ between locomotor categories at α = 0.05.

### 2.2 Pose spectrum analysis (2) and intraspecific scaling analysis (3)

Five brown rats (*Rattus norvegicus*, RjHan:WI strain), six domestic guinea pigs (*Cavia porcellus*), and two Patagonian maras (*Dolichotis patagonum*) were available. We chose these species as they exhibit a gradient both in cursoriality and BM (from the lightest, generalist rat to the heavier, semi‐cursorial guinea pig, to the relatively heavy, fully cursorial mara) (Elissamburu & Vizcaíno, 2004; Rocha‐Barbosa & Casinos, 2011; Seckel & Janis, 2008). We categorized guinea pigs as semi‐cursorial, as their limbs show generalized limb proportions (Elissamburu & Vizcaíno, 2004; Rocha‐Barbosa & Casinos, 2011; Seckel & Janis, 2008), yet are unable to supinate the hand (Buxton & Peck, 1989), which is a trait related to cursorial species (Gasc, 2001; Hildebrand, 1974).

The excised frontal limbs were fixed with tight straps in a spectrum of elbow flexion‐extension angles and supinated or pronated hand positions (Figure S2). This represents the maximum possible rotations within the elbow joints without (knowingly) damaging tissue. Then, the specimens were frozen and the procedure for quantifying the IJDs was done as described above in the *interspecific scaling analysis* (1). The resulting distances were each divided by the mHC of the measured limb to get body size independent results.

To investigate the *intraspecific scaling relationship* (3) of average IJD to BM, a separate regression on log‐transformed median IJDs and log BM for each species (rat, guinea pig, mara) was performed. As specified above in the *intraspecific analysis* (1), it was tested whether the slope deviated statistically from isometry.

### 2.3 Range of motion analysis (4)

Humeri, radii, and ulnae from collection material of the Museum für Naturkunde, Berlin, of *R. norvegicus* (specimen ID: ZMB 105825), *C. aperea* (ZMB 105757), and *D. patagonum* (ZMB 13323) were μCT‐scanned and 3D surface meshes created in Dragonfly. To reduce computational time, mesh sizes were decimated (Bishop et al., 2023): Articulation sites were selected using the lasso tool and evenly re‐meshed to 2000 to 5000 faces, depending on the complexity of the surface (e.g., long bones of rats and humeri in general required more faces to preserve the shape of the articulation site than the mara bones and radii, respectively). Distal epiphyses and the rest of the bone were reduced to roughly the same number of faces each as the articulation site, resulting in 6000 to 15,000 faces total. Articulation sites and the whole bones were exported as separate meshes in .obj format. Of the ulnae, the selected articulation site did not contain the radio‐ulnar articulation area, similarly to the *scaling analysis* (Figure 1, Figure S2).

**FIGURE 2:**
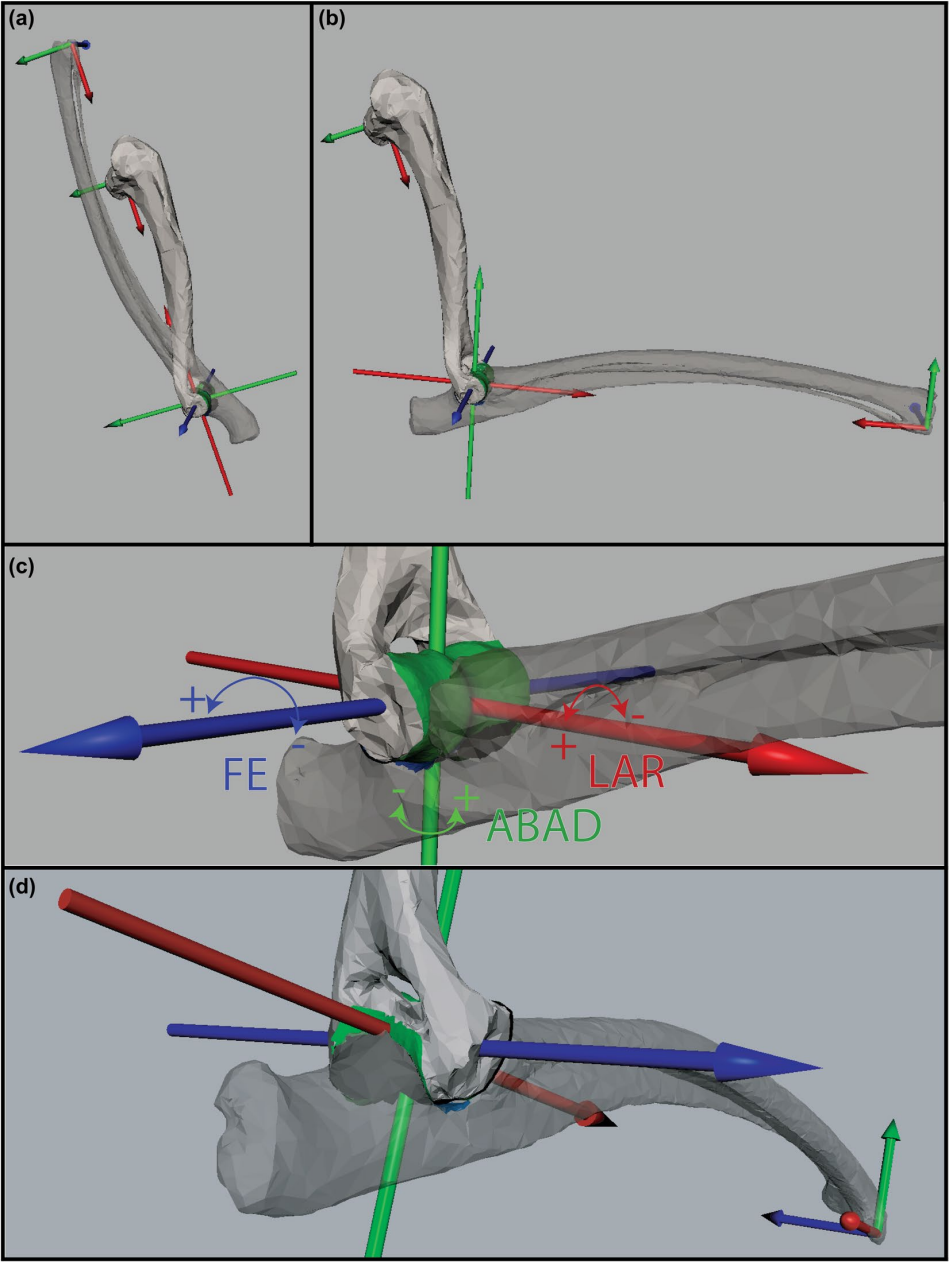
Exemplary reference pose of the right elbow of mara in lateral view. The axes of the cylinders that were fit in the ulnar and humeral articulation sites (see text for details) were aligned, resulting in a collapsed (non‐viable) reference pose with all translations and rotations set to zero (a). The center of rotation (COR) is located at the center of the blue, green, and red axes in the humeral trochlea/ulnar notch. (b) Partially extended humero‐ulnar joint (i.e., negative rotation about the blue axis) of −111°. (c) Magnified COR in latero‐cranial aspect at −111° extension. Biological meanings of rotations are color coded: Flexion‐extension (blue, FE), abduction/adduction (green, ABAD), and long axis rotation (red, LAR). To enhance visibility, the radio‐ulnar mesh is shown opaque, the humeral articulation surface is colored green, and the ulnar articulation site blue. (d) Same magnification as c in latero‐caudal aspect. Note that translations in the ROM analysis are measured and presented along the COR axes shown in a.

To ensure comparability of 3D pose results between species, a uniform reference pose (where all rotations and translations are set to zero) was constructed, following a previous protocol (Gatesy et al., 2022): In Geomagic Wrap spheres as geometric primitives were fit to the humeral head and ulnar styloid process, and cylinders in the humeral trochlea and ulnar notch. In the software Autodesk Maya (Version 2020, Autodesk Inc., San Rafael, CA, USA), the bone meshes and primitives were imported (Figure S3). An anatomical coordinate system (ACS) was constructed for humerus and ulna of each specimen separately. Each bone has proximally a mobile and distally a fixed ACS (ACSm and ACSf, respectively). In the humerus, a direct vector pointing from the center of the sphere matched to the humeral head towards the center of the cylinder in the trochlea created the X‐axis of the ACSm, and the longitudinal axis of the cylinder the Z‐axis of the ACSf. The other axes of the ACS are formed perpendicularly. In the ulna, the direct vector of the proximal cylinder (in the ulnar notch) and the distal sphere (in the ulnar styloid process) formed the X‐axis of ACSm. We deviated from the protocol that the Z‐axis of the distal ACSf was created with the longitudinal vector of the proximally fit cylinder. Ultimately, the position and orientation of the ulnar ACSm was matched to the humeral ACSf (Figure 2a) to form a joint coordinate system. In other words, the COR of the humero‐ulnar joint is located at the center of both the cylinders fit to the humeral and ulnar articulation sites. This reference pose was constructed in a right‐handed coordinate system with three perpendicular axes. The Z‐axes aligned to the humeral and ulnar medio‐lateral axes in the articulation sites. Therefore, Z‐rotations correspond to flexion and extension (FE, with 0° corresponding to a hyper‐flexed, and − 180° a fully extended elbow), Y‐rotations to abduction and adduction (ABAD, adduction positive, abduction negative), and X‐rotations to long axis rotation (LAR, inward rotation being positive) of the humero‐ulnar joint (Figure 2c). Translations are reported along the same axis, with the Z‐axis corresponding to medio‐lateral (laterally positive), Y‐axis to anterio‐posterior (posterior positive), and X‐axis to proximo‐distal translation (proximally positive) in the reference pose. Note however, that in hinge‐like joints biological meanings of the translations may shift depending on the joint's orientation (Manafzadeh & Gatesy, 2021). Finally, the mesh of the radius was positioned in a pronated position, based on size‐adjusted ex vivo CT scans of the same species from the *pose spectrum analysis* (*2*). The faces of the radius and ulnar mesh were connected at a small section along the shafts by a modelled bridge of polygons, creating a single radio‐ulnar mesh. This was needed to allow the use of the “APSE” algorithm (limited to the ROM analysis of two mesh entities). The radius is thus modeled as a passive element in this analysis (Richards et al., 2021). Meshes were exported from Maya and imported into Matlab for ROM analysis following the published protocol (Bishop et al., 2023).

The ROM analysis on rat, guinea pig, and mara was done using the recently published algorithm “APSE” (Bishop et al., 2023). This algorithm searches viable poses between 3D meshes of two bones, by rotating and translating the distal bone around and along an a priori defined COR. As this movement happens in 3D space, translations occur along and rotations around three axes, allowing movement with six DOF (Bishop et al., 2023; Manafzadeh & Gatesy, 2021; Richards et al., 2021). Any set of DOF coordinates is correspondent to a pose of the joint, which are visualized in a rotational (degree‐degree‐degree) pose space and a translational (mm‐mm‐mm) pose space (Bishop et al., 2023; Brocklehurst et al., 2025). The COR is thus the origin of the 6DOF coordinate system used to describe rotations and translations.

The search algorithm checks if the tested poses are viable by two exclusion criteria: Poses are deemed non‐viable if either the meshes of the bones *intersect* or are *disarticulated*. The *disarticulation criterion* is defined by a specified percentage of vertices of the distal articulation site exceeding an a priori set JS threshold.

Values of <50% overlap of articulation sites have been taken to indicate disarticulation in previous studies (Bishop et al., 2023; Brocklehurst et al., 2022; Fahn‐Lai et al., 2018; Jones, Brocklehurst, & Pierce, 2021; Kambic et al., 2017; Stevens & Parrish, 1999). Calculating the median IJDs in the *interspecific* (*1*) and *intraspecific scaling analysis* (*3*) allowed us to also set the *disarticulation overlap* threshold to 50%.

JS was estimated both from the interspecific (1) and the intraspecific scaling analysis (3). We measured the mHC of the specimens used in the ROM study, approximated the specimens' IJD and set it as JS threshold. For setup a_i_ (using the interspecific scaling as basis), the slope of non‐cursorial species informed the JS threshold of rats, while the slope of cursorial species informed the spacing threshold of guinea pigs and maras (see Results). The intraspecific scaling analysis (3) informed the other setups a–d (see below). This approach was chosen, as setups a–d investigate various restrictions of DOF. Here, the JS is thus based on ex vivo measures of the investigated species, opposed to IJDs estimated on a broad mammalian sample (1). This minimizes variance in estimated JS affecting the results between the setups.

Besides the viability criteria, “APSE” allows to set algorithmic search parameters (see Bishop et al., 2023, for details): *k* (broadly speaking both search speed and precision), *tolerance*, *w*, and *max generations* (all three defining when the algorithm terminates its search). After preliminary trials, we set *k* = 0.01, as higher values missed viable poses and lower values resulted in increased computational time without identifying new viable areas of the pose spaces. *Tolerance* was set to 1% in the 6DOF setups and to 5% with less DOFs (see below). *W* was set to 10 and *max generations* to 500.

Multiple ROM setups are tested here: setup a, allowing all six DOF; setup b, rotations about all three axes and translation in proximal‐distal direction (along the X‐axis), thus modelling 4 DOF; setup c, one rotation (flexion/extension) and translations along all three axes; and setup d, only rotations but no translations (see Herbst, Eberhard, et al., 2022; Herbst, Manafzadeh, & Hutchinson, 2022; Jannel et al., 2019; Merten et al., 2023; Richards et al., 2021; Wiseman et al., 2022). Additionally, we ran 6 DOF analyses where we increased and decreased the spacing threshold by 10% (setups a− and a+) and by using the interspecific scaling analysis to inform the JS (setup a_i_).

The 6 DOF setups (a, a+, a−, a_i_) were run with the set parameters described above. The other setups (b: 3Rot + TransX; c: 3Trans + RotZ; d: 3Rot) required limiting certain DOF, which were calculated in Matlab. For setup b, allowing translations only in the X direction, a position in which to “freeze” Z and Y translations needed to be found. We took the 6 DOF results from setup a and plotted the Z ~ Y translations of the viable poses. With the "boundary" function and a shrinkFactor = 1, a boundary was created. The centroid was calculated with the arithmetic means of each coordinate on the boundary line. The centroids’ coordinates were used as fixed translational values for the Z‐ and Y‐axes. Sometimes, the calculated centroid's position lay outside the alpha shape (see below) surrounding the viable poses found in setup a. Then, we used the mean and median Z and Y translation values instead, whichever was closer to the centroid. Setup c required the centroid of rotations along the Y ~ X‐axes; setup d required the centroid of all three translations. As the intelligent sampling of “APSE” applies a non‐exhaustive approach (thus, there is no guarantee of achieving uniform sampling across pose space), we regard a centroid calculation to be preferred to using mean or median values of all viable poses. “APSE” requires a certain range for each DOF, so we limited the range to 0.0001 mm for “frozen” DOFs.

The viable joint poses of each species (rat, guinea pig, and mara) and each of the ROM setups a, b, c, and d were interpreted using point cloud volume, in which an alpha shape is constructed around the viable poses in a pose space as tightly as possible without forming inner holes or excluding viable poses (Manafzadeh et al., 2021; Manafzadeh & Gatesy, 2020; Manafzadeh & Padian, 2018; Merten et al., 2023; Richards et al., 2021). We define the volume of the alpha shape as mobility based on previous studies (Brocklehurst et al., 2022, 2025; Lee et al., 2023; Manafzadeh et al., 2021; Manafzadeh & Gatesy, 2020; Merten et al., 2023; Richards et al., 2021). To compare rotational mobility and accounting for space distortion, FE rotations were cosine corrected before an alpha shape was constructed around the viable poses in the rotational pose space (Manafzadeh & Gatesy, 2020). It is good practice to use the same critical alpha value (the smallest alpha radius value which produces an alpha shape enclosing all the points) for all specimens (Manafzadeh & Gatesy, 2020; Manafzadeh & Padian, 2018; Merten et al., 2023). However, no consensual alpha radius value applicable for all species and setups could be found without restricting some results and being too permissive on others, given the differing ROM setups on multiple species. Consequently, the automatic critical alpha value was used. Therefore, the reported values should be treated with care and seen to display trends in differences between the observed species, rather than quantified results. Despite cosine‐correcting, the results are likely still affected by distortion through gimbal lock (see discussion by Lee et al., 2023). In our setup, gimbal lock occurs at values approaching −90° and 90° ABAD. Given the functional hinge‐like nature of the humero‐ulnar joint, with lower ranges in ABAD (see results), we have not corrected for gimbal lock distortion (Lee et al., 2023; Manafzadeh & Gatesy, 2020; Richards et al., 2021).

Besides rotational and translational mobility, maximum ranges in FE, ABAD, and LAR per setup (a, b, c, d) were compared in this study (Brocklehurst et al., 2025; Merten et al., 2023; Richards et al., 2021). Maximum ranges in FE were compared with previously published in vivo FE ranges reported on rats (Bonnan et al., 2016), guinea pigs (Rocha‐Barbosa et al., 1996, 2005), and maras (Vassallo & Rocha‐Barbosa, 2020). As Vassallo and Rocha‐Barbosa (2020) only report elbow extension angles in maras, flexion angles were assessed on publicly available images of grooming maras using the software Fiji (Schindelin et al., 2012). Bonnan et al. (2016) additionally report in vivo translational values in the humero‐ulnar joint during steady‐state locomotion. We used their reported translational values in setup d, not restricting the 3 rotational DOF and limiting the 3 translational DOFs according to the in vivo data. This way, we check whether “APSE” can approximate in vivo data and cross‐check the importance of translational DOFs.

All statistical analyses and creation of figures were done in Matlab (Version R2024b, Mathworks, Inc., Natick, MA, USA), Microsoft Excel (Version 16.93, Microsoft Corporation, Redmond, WA, USA), or R studio (Version 2023.12.1, Posit Software, PBC, Boston, MA, USA). As non‐native speakers, we used AI‐assisted tools to refine the language of parts of the final draft prior to submission.

## 3 RESULTS

### 3.1 Interspecific scaling analysis (1)

**FIGURE 3:**
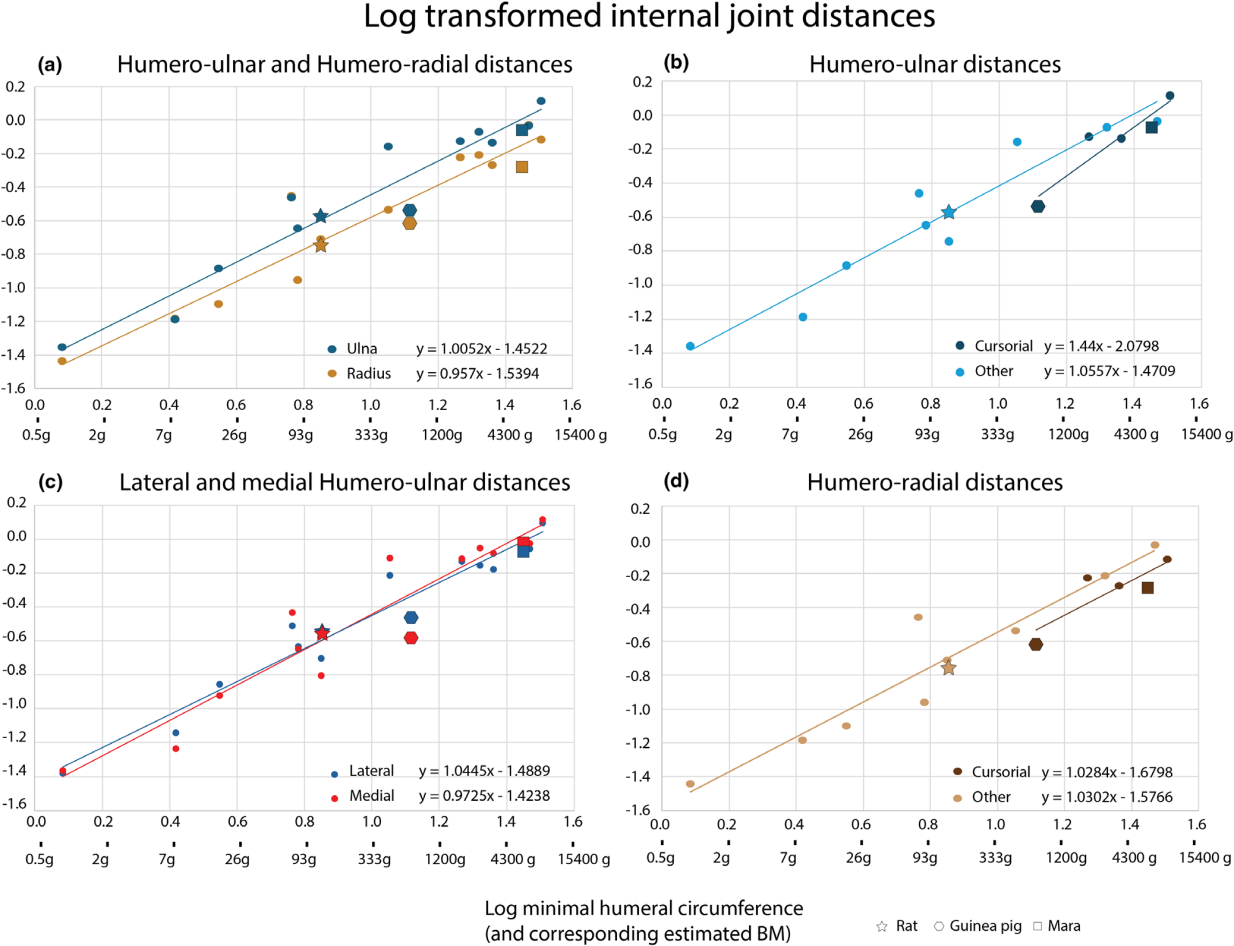
Log‐transformed results of the interspecific scaling analysis (1) of 15 species. The x‐axes show the log‐transformed mHCs (in mm) with the corresponding estimated BMs (Table 1) using the regression of mammals <20 kg BM by Campione and Evans (2012). Y‐axes show the log‐transformed median internal joint distances (IJDs). Slopes *b* < 1 correspond to negative allometry, *b* = 1 to isometry and *b* > 1 to positive allometry. Every setup shows isometric results, with humero‐ulnar distances in cursorial species showing the strongest tendencies towards positive allometry. Results for rat (star), guinea pig (hexagon), and mara (square) are highlighted.

BM estimations based on mHC are reported in Table 1 (Campione & Evans, 2012). The results of the *scaling analysis* are summarized in Table 2, Figure 3, and Figure S4, with some select data presented in Table S1. As the measured instances of IJDs (Figure 1d) form a plateau (Figure S1), we report the median distances, as they are less prone to outliers. In every regression, we found no significant phylogenetic signal within the residuals (*K* < 1, *p* > 0.05), summarized in Table S2.

**TABLE 2:** Measured median internal joint distances (IJDs). Bold rows refer to mean values, calculated from multiple specimens listed in the rows above. The mole's values are reported as in Table 1.

Common name	Estimated BM (g)	Median humero‐radial distance (mm)	Median humero‐ulnar distance		
			Whole	Lateral	Medial
Dog	17,006	1.08	1.43	1.32	1.47
Dog	11,327	0.71	1.68	1.70	1.67
Dog	2097	0.49	0.76	0.71	0.78
**Dog**	**10,143**	**0.76**	**1.29**	**1.24**	**1.31**
Guinea pig	695	0.24	0.29	0.34	0.26
Patagonian mara	5581	0.43	0.64	0.71	0.61
Patagonian mara	6226	0.60	1.08	0.98	1.27
**Patagonian mara**	**5903**	**0.52**	**0.86**	**0.85**	**0.94**
Cat	3349	0.52	0.62	0.61	0.66
Cat	3385	0.55	0.75	0.62	0.90
Cat	3340	0.54	0.79	0.75	0.89
**Cat**	**3358**	**0.53**	**0.72**	**0.66**	**0.82**
Eurasian otter	6696	0.92	0.92	0.87	0.94
Eurasian harvest mouse	8	0.06	0.06	0.07	0.05
Short‐tailed opossum	127	0.19	0.18	0.19	0.15
Banded mongoose	1837	0.59	0.74	0.74	0.77
House mouse	18	0.07	0.13	0.14	0.12
Brown rat	128	0.17	0.26	0.28	0.27
Tufted capuchin	2581	0.61	0.85	0.70	0.88
Red squirrel	467	0.28	0.69	0.61	0.77
Etruscan shrew	1	0.03	0.04	0.04	0.04
European mole	645 (73)	0.35	0.34	0.30	0.37
Swinhoe's striped squirrel	82	0.11	0.22	0.23	0.22

#### 3.1.1 Humero‐ulnar IJDs

The median humero‐ulnar distance increases linearly with the mHC (*b* = 0.0343), with little difference between the medial (*b* = 0.0361) and lateral (*b* = 0.0321) IJDs. When only non‐cursorial species are considered, the increase in IJD is lower (*b* = 0.0341) compared with (semi‐) cursorial species (cat, dog, guinea pig, mara, and mongoose) (*b* = 0.0443) (Figure S4). When IJD and mHC are log transformed (Figure 3), the ulnar distances increase isometrically (*b* = 1.0052 ± 0.174), also when medial and lateral articulation areas are considered separately (medial: *b* = 1.0445 ± 0.207; lateral: *b* = 0.9725 ± 0.136). Only considering non‐cursorial species, the scaling is isometric (*b* = 1.0557 ± 0.256), with a non‐significant (*p* = 0.3798) tendency towards positive allometry in the subset of (semi‐) cursorial species (*b* = 1.440 ± 0.956).

#### 3.1.2 Humero‐radial joint

In general, we observe smaller IJDs in the humero‐radial joint compared with the humero‐ulnar joint (Figure S4), with a lower slope (*b* = 0.0245). The slope is also lower in the radii of (semi‐)cursorial species (*b* = 0.0204) compared with non‐cursorial species (*b* = 0.0309) in our dataset. In the log‐transformed data (Figure 3), we observe isometric scaling across all species (*b* = 0.957 ± 0.18), as well as within non‐cursorial species (*b* = 1.0302 ± 0.269) and (semi‐)cursorial species (*b* = 1.0284 ± 1.163). The groups do not differ statistically (*p* = 0.9969).

### 3.2 Pose spectrum analysis (2)

#### 3.2.1 Humero‐ulnar joint

**FIGURE 4:**
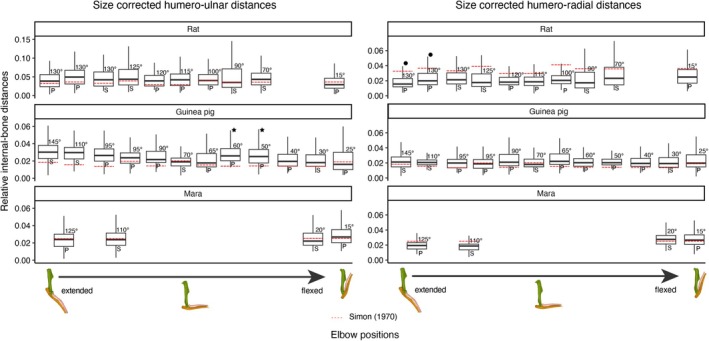
Boxplot of size corrected internal joint distances (IJDs) in a pose spectrum. Each boxplot represents one elbow of one specimen; outliers are not depicted in the graph. Size corrections were achieved by dividing each measure of IJD by the specimens' minimal humeral circumference (mHC). Red stripes show the predicted mean IJD based on each specimens' predicted BM (by mHC, see text for details) and the BM's reported correlation to cartilage thickness by Simon (1970), with the predicted IJD being twice the predicted cartilage thickness. Images below the graph display the range of extended poses from 130° to 15° on bones of rats, with humerus in green, ulna in brown, and radius in pink. On top of each boxplot is the degree of extension indicated in degrees. P and S refer to pronated and supinated postures, respectively. Note the different scaling in the rat humero‐ulnar results, to account for the differences to the other species in relative distance. Boxplots marked with a star refer to similar setups from a single specimen of guinea pig. This individual appears to have slightly larger IJDs than others. The black dots represent a similar flexion angle and hand position from two specimens of rats, also indicating variation in joint distances between the individuals.

Overall, the median IJD in the humero‐ulnar joint is larger in rats than guinea pigs and maras when corrected for body size (Figure 4). In absolute terms, maras show the largest median distances (0.75 mm), followed by rats (0.38 mm) and guinea pigs (0.35 mm) (Figure S5). Differences in IJDs along the flexion‐extension gradient display a mosaic pattern within each species. The rats' and guinea pigs' flexed poses exhibit marginally lower distances than extended poses, but individual differences between the specimens become apparent (Figure 4). In maras, the median IJD is unaffected by limb pose.

#### 3.2.2 Humero‐radial joint

IJDs in the humero‐radial joint show no differences between the species when corrected for body size (Figure 4). In absolute terms, maras have the largest median distances (0.67 mm), followed by guinea pigs (0.28 mm) and rats (0.20 mm) (Figure S5). Both rats and maras display higher distances in flexed opposed to extended elbows. Pronation and supination appear to have no effect on median IJD.

### 3.3 Intraspecific scaling analysis (3)

#### 3.3.1 Humero‐ulnar joint

Across the exemplar species, we report isometric scaling within the humero‐ulnar joint (Figure S6), albeit with positive allometric tendencies.

In rats, the slope (*b* = 1.1282 ± 1.2073) is less steep than in guinea pigs (*b* = 1.5683 ± 2.0278) and maras (*b* = 1.4893 ± 7.0908) with log transformed data.

#### 3.3.2 Humero‐radial joint

Across all species, we report isometric scaling within the humero‐radial joint (Figure S6).

However, Rats display negative allometric tendencies (*b* = 0.0254 ± 1.0615), while guinea pigs (*b* = 1.4853 ± 0.5309) and maras (*b* = 6.0507 ± 6.0428) display positive allometric tendencies with log transformed data.

### 3.4 Range of motion analysis (4)

**FIGURE 5:**
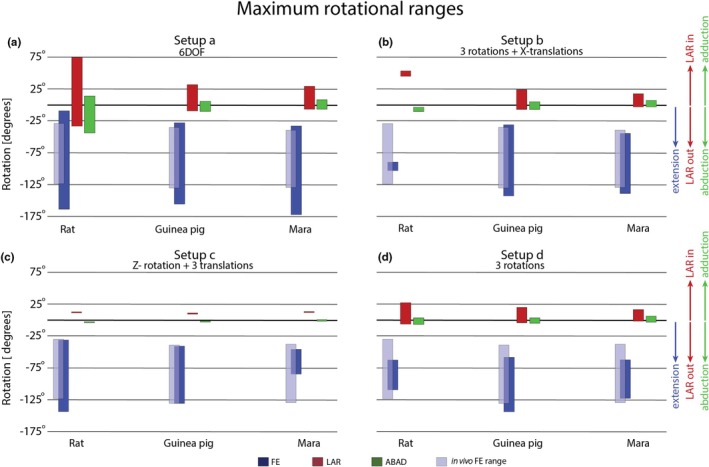
Maximum ranges of rotational DOF between the various setups. Blue bars represent flexion‐extension (FE), with semi‐transparent blue bars referring to estimate in vivo FE ranges of each species based on published records (Bonnan et al., 2016; Rocha‐Barbosa et al., 1996; Rocha‐Barbosa et al., 2005; Vassallo & Rocha‐Barbosa, 2020, see text for details). (a) Setup a, all six DOF were allowed freely, thus three rotational and three translational DOF. (b) Setup b, the three rotations were allowed freely, translation only along the X‐axis. (c) Setup c, rotations were limited to FE and all three translations were allowed freely. (d) Setup d, only rotations were allowed freely. ABAD, abduction/adduction; LAR, long axis rotation.

The maximum ranges of each rotational DOF differ substantially between the varying setups, generally decreasing when DOFs were restricted in the analysis (Figure 5, Figure S7). The amount of reduction varies between species and DOF considered. While there are differences present in FE between the three exemplar species (rats, guinea pigs, maras) when 6DOF (setup a) were allowed, the largest difference occurs in LAR and ABAD (Figure 5a): Generally, maximum flexion is lower, and LAR and ABAD are generally more restricted in the more cursorial guinea pig and mara. An increase and decrease of JS (setups a+ and a−) resulted in slight changes of maximum range in each DOF (Figure S7).

**FIGURE 6:**
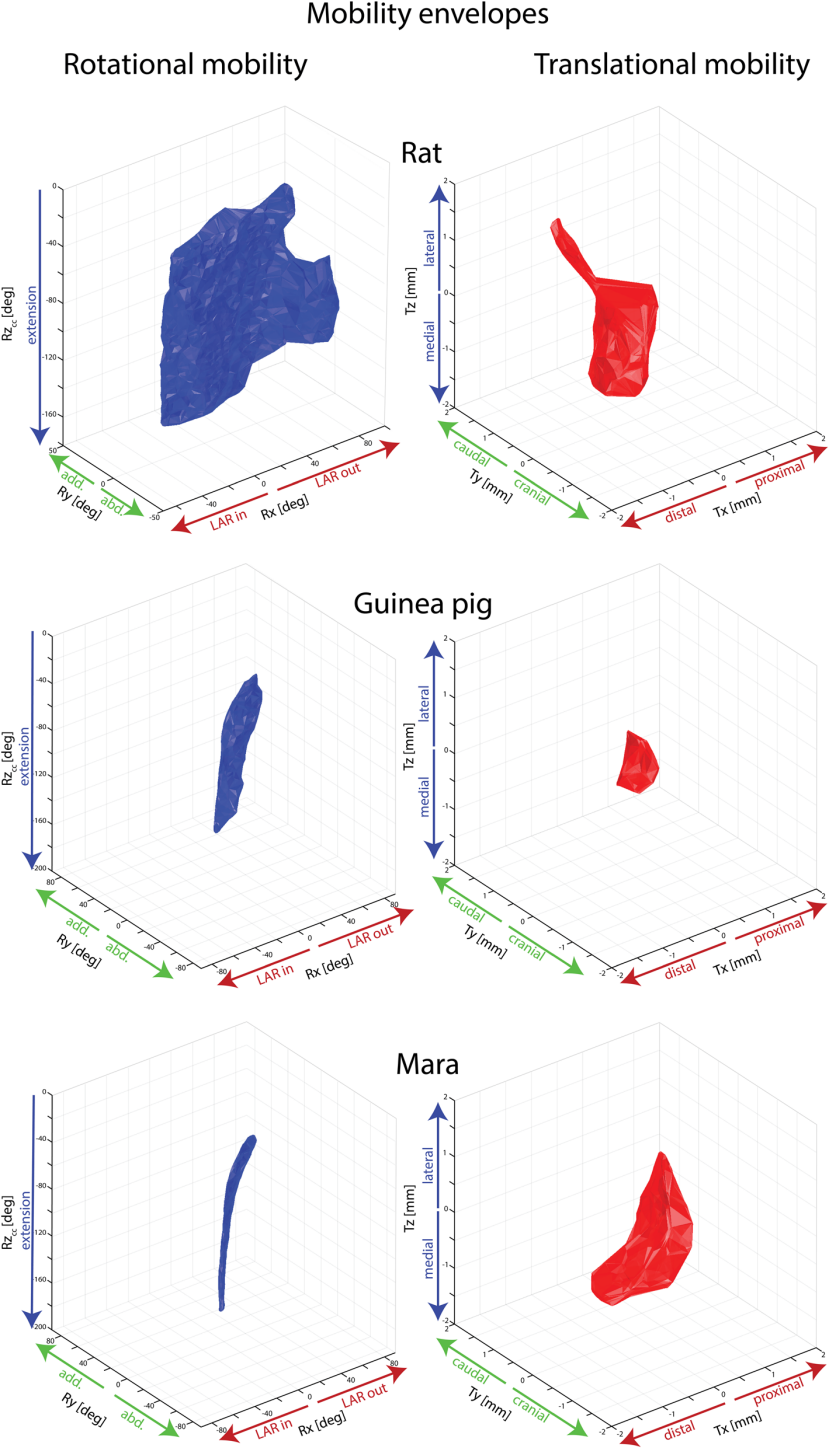
3D representation of the ROM analysis setup a. The left graphs (blue) show the alpha shape around rotational values of the viable poses in a deg‐deg‐deg space; separate for rats, guinea pigs, and maras. Each axis corresponds to one rotational degree of freedom (Rx‐ LAR, Ry‐ ABAD, Rz_cc_‐ FE). The FE values have been cosine corrected (Manafzadeh & Gatesy, 2020). The right graph (red) shows the alpha shape around the translational values of the viable poses. Here, each axis corresponds to one translational degree of freedom in mm (Tx, Ty, Tz). Note that translations are along the axis of the reference pose (Figure 2a), and biological meanings of translations may change depending on the joints' rotation (Manafzadeh & Gatesy, 2021). In this graph, all alpha shapes are created with an alpha radius of 10 for rotations and 1 for translations. File S1 provides interactive 3D graphs.

Rotational and translational mobility (in deg^3^ and mm^3^, respectively) differs substantially between species and setups (Table 3, Figure 6). Generally, an increase in cursoriality and/or BM is reflected in a decrease of mobility. Also, in all species, a decrease in mobility is present when the number of DOFs is reduced. We find it noteworthy that maras and rats display a similar translational mobility despite a near ten‐fold difference in BM and size. It implies that the rats' humero‐ulnar joint achieves mobility by allowing relatively larger translations. Rotational mobility in rats was lower in setup b than in setup d (see discussion), but this is the exception to the general decrease in setups with fewer DOFs. In setup a− (a 10% decreased JS compared with setup a) both rotational and translational mobility decreased, while an increase by 10% (setup a+) resulted in increased mobility in guinea pigs and rats, but somewhat surprisingly a slight decrease in rotational mobility in maras (Figure S7). In setup a_i_, the JS lay generally between a+ and a−, with the resulting mobility also displaying an intermediate range. Only in mara, the a_i_ distance was larger than a+ and concordantly the approximated mobility increased. However, it was still lower than the rat and guinea pigs' mobility, further highlighting the general trend of decreased mobility with increasing cursoriality and BM.

**TABLE 3:** Overview of the mobility (volumetric) comparison of the ROM analysis.

Species	Rat							Guinea pig							Mara						
ROM setup	a	a−	a+	a_i_	b	c	d	a	a−	a+	a_i_	b	c	d	a	a−	a+	a_i_	b	c	d
Joint spacing (JS) [mm]	0.3822	0.3440	0.4204	0.3530	0.3822			0.2712	0.2441	0.2983	0.2958	0.2712			0.5882	0.5294	0.6470	0.9003	0.5882		
Rotational mobility [deg^3^]	102,174	52,670	126,987	52,565	102	251	2695	11,036	10,056	16,000	15,239	5143	130	2291	6491	5051	7109	9872	2522	79	1028
Translational mobility [mm^3^]	0.692	0.329	0.907	0.364	0.00	0.117	0.00	0.082	0.060	0.104	0.099	0.000	0.028	0.00	0.668	0.486	0.832	1.247	0.000	0.220	0.00
Rotational mobility comparison to setup a [%]	/	52	124	51	0	0	3	/	91	145	138	47	1	21	/	78	110	152	39	1	16

*Note*: Each column presents the results of one ROM setup, separate for the three investigated species. Setup a allowed free rotations and translations. Setups a− and a + allowed the same DOFs, but the spacing threshold between humerus and ulna was reduced or increased by 10%, respectively. In setup a_i_ the joint spacing threshold was calculated based on the *interspecific scaling analysis* (*1*). Here, the regression slope of non‐cursorial species informed spacing for rat, the slope of cursorial species for guinea pigs and maras (Figure 3). All other setups (a, a+, a−, b, c, d) based the spacing threshold on the *intraspecific scaling results* (*3*). Setup b allowed for three free rotations and free translation only on the X‐axis (see Figure 2 and text for details). Setup c allowed for free translations, but restricted rotations on the X‐ and Y‐axes (LAR and ABAD), only allowing free Z‐axes rotations (FE). Setup d only allowed rotations, preventing any translations. The 4th row presents rotational mobility (the volume of the alpha shape around the viable poses in the rotational pose space in deg^3^). The 5th row presents the translational mobility (volume of the alpha shape fitted around the viable poses in the translational pose space in mm^3^). The bottom row compares each column's rotational mobility to the rotational mobility of setup a.

## 4 DISCUSSION

We investigated how internal joint distances (IJDs) scale in the humero‐ulnar and humero‐radial joints of small‐ to medium‐sized mammals. Using ex vivo CT scans of 15 species (estimated body mass of individuals ranged from 1 g to 17,006 g), we measured ~15,000–20,000 evenly spaced distances across the articular surfaces of each specimen (*1*). To assess the influence of limb poses on IJDs, we analyzed a subset of three rodent species (rats, guinea pigs, maras) in a spectrum of poses—flexion to extension and pronation/supination (*2*). Within these exemplar species we also conducted a scaling analysis at the *intraspecific level* (*3*).

Because estimates of JS and restrictions in DOF directly affect modeled ROM of a joint (e.g., see Arnold et al., 2014; Demuth et al., 2020; Hutchinson et al., 2005; Jones, Dickson, et al., 2021; Lee et al., 2023; Manafzadeh & Gatesy, 2021; Regnault & Pierce, 2018; Richards et al., 2021; Wiseman et al., 2022), we also performed a ROM analysis of the humero‐ulnar joint in the three rodent species (*4*). To ensure biologically meaningful and comparable outcomes, we report the methodology and results from multiple ROM setups.

### 4.1 Interspecific scaling of internal joint distances (IJDs)

Previously published studies investigating cartilage thickness across ranges in mammalian BMs reported positive allometry underneath the patella, and in the hip, shoulder, elbow, and knee joints (Simon, 1970; Stockwell, 1971), but also negative allometry in the knee joint (Malda et al., 2013). Our results in the elbow joints showed isometric scaling in IJDs. With these results appearing to contradict each other, it is necessary to take a closer look at the differences in sampled species and joints in the studies.

We believe that the locomotor type of a species and a size constraint on cartilage thickness largely explain the observed patterns. Cursorial species are adapted for fast and/or sustained locomotion (Clifford, 2010; Gasc, 2001; Hildebrand, 1974; Stein & Casinos, 1997). Fast locomotion is expected to increase absolute forces acting on the limb bones (Biewener & Patek, 2018; Hildebrand, 1974; Villacís Núñez et al., 2022; Warner et al., 2013), which in turn theoretically require thick, robust cartilage to maintain biomechanical resistance to compression forces (Kiviranta et al., 1987; Malda et al., 2013). The isometric relationship in humero‐ulnar joint distance reported here shifted towards a positive allometric tendency when only cursorial species were considered. Since Simon (1970) investigated the thickest cartilage section of each joint, they may have sampled regions especially required to withstand compression forces. Further, in their limited sample, the larger species were all cursorial (dog, sheep, and the arguably graviportal cow), while the smaller species were the non‐cursorial mouse and rat. The reported positive allometry thus may present a bias towards withstanding high forces in cursorial species. Stockwell (1971) sampled cartilage thickness in the distal femur, also focusing mostly on cursorial species (cow, sheep, cat, dog, rabbit, and human; and non‐cursorial rat and mouse). Again, the sampling bias towards cursorial species may have shifted the scaling towards positive allometry. To maintain safety factors, mammals with higher BMs reduce forces acting within the limbs by an upright posture, aligning the limbs more in the direction of acting forces (Biewener, 2005; Biewener & Patek, 2018; Vassallo & Rocha‐Barbosa, 2020; Warner et al., 2013). This effect is less present in closely related species (Biewener & Patek, 2018; Day & Jayne, 2007). Since cursorial species tend to have similar, more upright postures (Biewener & Patek, 2018; Gasc, 2001; Hildebrand, 1974; Pike & Alexander, 2002), forces likely increase here within the bone, necessitating positive allometric tendencies in cartilage thickness.

By contrast, Malda et al. (2013) sampled cartilage thickness in the femoral condyles of 58 species, ranging from 25 g (mouse) to 4,000,000 g (African elephant). They report negative allometry in cartilage thickness; thus, heavier species have relatively thinner cartilages, a trend which is supported by measures of the sub‐articular distal humerus across a range of mammalian body masses (Bonnan et al., 2013). These results are apparently in stark contrast to the results of Simon (1970) and Stockwell (1971), reporting positive allometry, and slightly to this study where we report isometry. Malda et al. (2013) conclude that diffusional constraints limit cartilage thickness in mammals. They argue that adult articular cartilage lacks vascularization and that too thick cartilages cannot be supplied and maintained. This, in turn, results in negative allometry when very large species are included in the sample. In a subsample of data from Malda et al. (2013) only including specimens below 17,000 g (matching the sampling range of this study), isometric scaling in femoral cartilage thickness is present (Figure S8), aligning with the results presented here. Concerning very large tetrapods, evidence of vascular channels in some non‐avian dinosaurs is present, supporting the presence of potentially thicker cartilages in their limb bones (Bonnan et al., 2013; Holliday et al., 2010). Malda et al. (2013) report a relatively constant static compressive stress on cartilage tissue across the mammalian BM range, facilitated by increased congruence and articular surface area (Bonnan et al., 2013; Malda et al., 2013), and adjustments of posture and activity in larger species (Biewener & Patek, 2018; Gasc, 2001; Hildebrand, 1974; Pike & Alexander, 2002). This allows larger species to have functional, yet relatively thinner cartilage. We propose that this cartilage thickness constraint acts on species larger than sampled here (thus beyond 11 kg, which was the mean BM of our largest species) and would thus not affect the majority of terrestrial mammals (modal BM of 100 g, see Brown et al., 1993; Lovegrove, 2001; Lovegrove & Haines, 2004).

We acknowledge that our methodology of measuring IJDs between the adjacent bones does not discriminate between potential differences in cartilage thickness of each adjacent bone. This may however be the case, as has been reported in the human hip, where acetabular cartilage is reportedly thinner than cartilage at the femoral head (Shepherd & Seedhom, 1999). To our knowledge, experimental data are scarce and not available for the bones of the elbow joint.

Differences in cartilage thickness between bones of a single joint (Shepherd & Seedhom, 1999) and across different joints within the same species (Stockwell, 1971) have been reported. It is therefore plausible that the isometric scaling reported for the elbow joints here and the negative allometric scaling within the knee joint (Malda et al., 2013) could be related to differing cartilage thickness dynamics between the two joints examined. Comparison between the here presented results of elbow IJDs and knee cartilage thickness yields different results for humero‐ulnar and humero‐radial joints (Table S1). The halved median humero‐ulnar distances of mouse, rat, and dog reported here are only slightly smaller than the reported cartilage thickness of the same species' femoral cartilage thickness, but the specimens were also of lower BM. One dog examined here with BM = 11.3 kg showed halved humero‐ulnar distances of 0.843 mm, very similar to 12 kg dogs from Malda et al. (2013) with a medial femur cartilage thickness of 0.849 ± 0.185 mm. Thus, the ulnar cartilage thickness seems comparable with the cartilage of femoral condyles, at least in mouse, rat, and dog, where data are available. The approximated radial cartilage thickness is clearly lower, though. In fact, for some species, we found similar distances in radii and ulnae (otter, harvest mouse, short‐tailed opossum, mole) while others have much larger values in the humero‐ulnar IJDs (dog, mara, cat, squirrels). The arithmetic mean across our whole sample reports the humero‐ulnar IJDs to be 1.4 times larger than the humero‐radial joint distances (also see discussion on intraspecific scaling and force transmission within the joints below). Given the similarities between median humero‐ulnar IJD and femoral cartilage thickness, and isometric scaling in small specimens of Malda et al. (2013), it is thus likely that the negative allometric relationships in the humero‐ulnar joint are due to a size constraint. This may still be also true for the radius, but the direct comparison between femur and radius is not as clear.

Smaller IJDs were present in cursorial species both in the humero‐ulna and the humero‐radius joint (Figure 3). We conclude that this result is a sign of higher congruency, simply with more surface area being close to the apposing articulation surface. This was proposed to be an adaptation of joints to high BMs (Bonnan et al., 2013; Malda et al., 2013; Simon, 1970). While this is concordant with the fact that cursorial mammals tend to be generally larger (Alexander & Jayes, 1983; Carrano, 1999; Lovegrove & Haines, 2004), we acknowledge our small sample size (*n* = 5) affecting the results.

### 4.2 Limb poses do not affect internal joint distances (IJDs)

We found a mosaic pattern in how the flexion/extension angle affects the IJDs. For example, the humero‐ulnar distances decrease slightly in more flexed elbows, especially of guinea pigs. Controversially, the humero‐radial distances increase in flexed elbows of rats and maras. We believe that our definition of the humeral articulation site is causing this discrepancy. In extended poses, the ulnar olecranon process slides into the olecranon fossa of the humerus. However, the area which we define as the articulation site of the humeral trochlea does not extend that deeply into the olecranon fossa. In fact, there is usually a clear edge visible, which we define as the end of the trochlear articulation site, and the beginning of the olecranon fossa. In extended poses, the vertices of the ulnar articulation site closest to the olecranon process are thus located away from the edge of the humeral articulation site when it enters the olecranon fossa. This increases the overall humero‐ulnar distance. Similarly, in flexed positions, the radius slides into the radial fossa of the humerus. Again, the anterior part of the radial head is then located away from the edge of the humeral articulation site, increasing the overall distance slightly compared with less flexed poses.

These differences are, however, relatively minor, as the difference between individual specimens appears to have a bigger effect. We found occasionally bigger differences between trials of similar flexion angles from different specimens (e.g., see the boxplots marked with a dot and stars in Figure 4). Also, as this study aims to be applicable for fossil specimens when no in vivo data are available, our definition of humeral articulation site follows previous applications (e.g., Bishop et al., 2021, 2023; Brocklehurst et al., 2025). We thus conclude that positioning of the elbow has a negligible effect on the distances between the articulation surfaces of the bones.

### 4.3 Intraspecific scaling of internal joint distances (IJDs)

We report isometric scaling within single species across all setups (Figure S6). This is in contrast with a previous study reporting positive allometry within mature bovine and ovine tibiofemoral cartilage yet corresponds to the finding of isometry in immature porcine specimens (McLure et al., 2012). Relatively thicker cartilage is argued to be an adaptation to increased loading (see discussion above), but more comparative data are needed. Species with higher BMs maintain safety factors by adopting a more upright posture, aligning the limbs closer to the direction of acting forces. In closely related species, and consequently intraspecifically, fewer postural adjustments are present (Biewener & Patek, 2018; Day & Jayne, 2007), indicating absolute higher acting forces in heavier specimens of the same species.

Although McLure et al. (2012) do not report BM of the investigated specimens, it is safe to assume the ovine, bovine, and porcine specimens reach higher BM than our sample of rats, guinea pigs, and maras. We report a higher tendency towards positive allometry in the two heavier species (guinea pig, mara). We propose that intraspecifically positive allometry is thus required in cartilage thickness of heavier species than sampled here.

Further, we report tendencies towards positive allometry in the ulnar and negative tendencies in the radial joint in rats. Ex vivo data on dogs report an almost equal force transmission from radius and ulna to the humerus (Mason et al., 2005). In vivo, when the ulna acts as a lever for the anti‐gravity muscle triceps brachii (Milne, 2016), one can assume higher forces acting through the proximal ulna than the radius. This effect may even be stronger in less cursorial species than dogs, where the radius can rotate freely around the ulna (Gasc, 2001; Hildebrand, 1974). Here, the ulnar proximal load likely increases, as was measured ex vivo in human forearms (Pfaeffle et al., 2000). Thus, possibly intraspecific positive allometry in cartilage thickness is required in load‐bearing joints, which we argue is in rats the proximal ulna opposed to the radius. However, more research is needed to illuminate that relationship and the effect of locomotor type, posture, and load bearing on the cartilage thickness in (elbow) joints.

### 4.4 Joint spacing (JS) and its effect on ROM analyses

The ROM analysis of the elbow in rats, guinea pigs, and maras confirmed previous results of a strong coupling between rotations and translations in the mammalian elbow joint. The strongest interactions have been previously recorded between LAR and translation corresponding to the Y‐axis in our setup, in opossums (Brocklehurst et al., 2025). Further, strong coupling between LAR and translation in the medio‐lateral direction in Echidnas with a sprawling posture is reported (Regnault et al., 2021), the Z‐axis here. In this study, we report the largest range in translation along the Z‐axis (Figure 6). But generally, exclusion of any translational DOFs falls short of approximating in vivo data on rotational ranges which is available for rats, guinea pigs, and maras (Figure 5). Previous studies on guinea pigs and maras focused on 2D FE angles. While FE angles obtained in 3D setups are not directly comparable with 2D FE angles obtained from a lateral view (Robertson & Gordon, 2004), they illuminate the trend that exclusion of DOF can lead to biologically unrealistic results.

Bonnan et al. (2016) measured in vivo 3D kinematics in rats with a comparable zero‐pose setup to ours. The reported FE ranges were not reached in our setups b and d (Figure 5). The authors report “tiny” translations in the elbow joint, implying that translations contribute minimally to FE during steady state locomotion in rats. However, exclusion of translations in setups b and d resulted in smaller rotational ranges than reported in vivo. In fact, when we included Bonnan et al.'s (2016) reported translational values in setup d (Figure S7), we were able to approximate the reported values in FE, indicating the importance of translations even when they appear insignificant. The remaining difference may be due to slightly varying zero poses with a different approximated COR position and no adjustment in allowed JS, despite a likely difference in rat BM compared with the in vivo data (Bonnan et al., 2016). The setup b ranges appear extremely low; the results are thus somewhat contrary to previous studies which indicate that inclusion of a single translation in distraction–compression direction of a joint may yield results comparable with in vivo data, while maintaining meaningful ROM estimations (Manafzadeh & Gatesy, 2021). Manafzadeh and Gatesy (2021) set translational limits based on ex vivo translational values, which were not available here and would not be available in paleobiological research. Further, the authors adjusted the reference pose setup, ensuring biological consistent interpretations of translations through varying rotations. The unrealistic ranges in rats setup b are likely the result of placement of the COR in the humero‐ulnar joint, which were based on the resulting centroid of translations along the Z‐ and Y‐axes of the setup a analysis. Our results show that this approach can lead to biologically unrealistic results in some species (rats and, to lesser degree, maras), but more realistic in others (guinea pig). However, it remains to be tested if our methodology is less realistic than previous applied methods in COR placement (e.g., Bishop et al., 2021; Brocklehurst et al., 2022; Hutchinson et al., 2005; Richards et al., 2021). This could possibly be done by investigating the position of COR on ex vivo or in vivo X‐ray data (Demuth et al., 2025). Additionally, it remains to be tested whether the inclusion of a prism, to ensure consistent meaning of translations along the X‐axes (see Manafzadeh & Gatesy, 2021, for details), or target‐distance based approaches with dynamic translations (Demuth et al., 2025; Lee et al., 2023) would change the results towards more biological realistic ranges in setup b.

Generally, all setups approximate previously published in vivo results of guinea pigs (Rocha‐Barbosa et al., 1996, 2005). Only in setup d, the maximal flexion angle does not reach in vivo reports, which is likely a result of COR positioning, as the general FE range is similar to in vivo data.

Publicly available images of self‐grooming maras show elbow flexion of ca. ‐45° (pers. obs. AS), while Vassallo and Rocha‐Barbosa (2020) report extension angles of −128° (the values are converted to be equivalent to our zero‐pose setup). These FE values were not reached in setups c and d.

Overall, our comparison of rotational ranges to in vivo data indicates a strong rotational‐translational coupling in the elbow joint (Brocklehurst et al., 2025; Regnault et al., 2021; Richards et al., 2021) and underscores the necessity to include all six DOF (Brocklehurst et al., 2025; Demuth et al., 2025; Manafzadeh & Gatesy, 2021; Regnault et al., 2021; Richards et al., 2021; Wiseman et al., 2022). When DOF are excluded, the positioning of the COR has a strong impact and needs to be biologically informed (Manafzadeh & Gatesy, 2021; Regnault et al., 2021; Regnault & Pierce, 2018, but see Demuth et al., 2025).

Interpreting the ranges of each rotational DOF in setup a is challenging, as all three species displayed overlapping results in FE. For example, while maximum flexion values in rats and maximum extended values in maras were higher than in the other species, a clear trend between FE ranges and BM or locomotor type was not apparent in our sample. ABAD and LAR ranges showed differences between locomotor types, however, with the (semi‐)cursorial guinea pig and mara displaying smaller ranges and therefore a more hinge‐like elbow function. An even more generally applicable approach is the quantitative comparison of rotational mobility (in deg^3^) (Manafzadeh & Gatesy, 2020). Here, we found a clear decrease in overall mobility from the generalist, light‐weight rat to the cursorial and heavier mara (Table 3). The guinea pig was closer to the maras' values, again indicating more hinge‐like elbow function in these two species. Comparing rotational mobility, rather than rotational range, clarified more the effect of JS settings on ROM results, with the smaller species being more affected by in‐ and decreases of JS (Table 3).

Overall, our results reinforce previous work that reliable JS estimation is important for reconstruction of ROM (Brocklehurst et al., 2022; Hutchinson et al., 2005; Jones, Brocklehurst, & Pierce, 2021; Lee et al., 2023; Manafzadeh et al., 2021). The estimated mobility varied greatly between the chosen spacing thresholds in setup a, a+ 10, a− 10, and a_i_, each allowing all 6 DOF. In comparative terms, however, the estimated spacing had limited effect on interspecific mobility patterns, as mobility consistently decreased from rats to guinea pigs to maras across all spacing threshold estimations. It has been argued before that the effect of estimated JS is small compared with the effect of bone shape if species of various locomotor types or BMs are compared (Lee et al., 2023, this study). This might be especially valid when multiple joints, antagonist muscle moment arms, and external forces acting on the limbs are modelled (Bishop & Pierce, 2024).

Visual comparison of the alpha shape in rotational pose space shows a clear difference between the three species examined (Figure 6). A clear restriction in ABAD and LAR in guinea pigs and maras was apparent. This was to be expected in cursorial species (Gasc, 2001; Hildebrand, 1974) as locking the elbow joint to function effectively in a single axis is concurrent with reducing stress on bones (Biewener & Patek, 2018; Fischer & Blickhan, 2006; Gasc, 2001; Hildebrand, 1974; Nikolai & Bramble, 1983) and is expected to reduce the variance of direction from which these stresses are experienced (Amson et al., 2017; Mielke & Nyakatura, 2019). Recent studies indicate ex vivo (Brocklehurst et al., 2025), in vivo (Scheidt et al., 2022), and theoretically (Richards et al., 2021) a deviation from a single axis function of the humero‐ulnar joint, at least in some species and to some degree.

Translational mobility (in mm^3^) was larger in rats than in the other two species and slightly larger than measured in the mara joint, despite the mara having 10‐fold its BM (Table 1). Concordantly, excluding translations from the ROM analysis had the biggest impact on the rats' rotational ranges and its rotational mobility. Rats appeared to reach rotational ROM by allowing relatively larger translations and displaying relatively higher IJDs (Figure 4) than guinea pigs and maras. Comparative data are scarce, but, in a broader phylogenetic context, it has been reported that tegu lizards allow relatively higher translations than opossums in their shoulder joint, reaching similar ex vivo rotational values (Brocklehurst et al., 2025).

While including translational DOF in ROM studies is crucial to achieve meaningful, biologically realistic results (see above), comparing translational mobility across species has to our knowledge not been included previously. Our research indicate informative results in translational mobility when comparing ROM in species with various locomotor specializations. Ultimately, with this limited sample, the effect of locomotor mode and BM on humero‐ulnar ROM cannot be conclusively disentangled, however. Future studies should aim at a broader sampling including multiple locomotor types across a wide range of BMs (Lee et al., 2023; Merten et al., 2023). Our results tentatively suggests that the degree of cursoriality accounts for the observed differences in mobility, as the BM of rats and guinea pigs is similar.

## 5 CONCLUSION

Internal joint distances (IJDs) in the humero‐ulnar and humero‐radial joints scale isometrically in small‐ to medium‐sized mammals. However, in cursorial species, these distances scale with positive allometric tendencies, despite exhibiting smaller IJDs overall. Our sampling also suggests that size constraints on cartilage thickness may only affect larger mammals (>11 kg), leading to negative allometry when larger species are included in the dataset (Malda et al., 2013). Additionally, we observed variation in IJDs between the humero‐ulnar and humero‐radial joints of the same species, likely reflecting intraspecific differences in cartilage thickness (Stockwell, 1971). The median humero‐ulnar IJD aligns with reported measurements of femoral condyle cartilage thickness (Malda et al., 2013). Limb poses had negligible effects on IJDs. We report isometric results in intraspecific scaling, somewhat contrarily to previously conducted research (McLure et al., 2012). We conclude that absolute higher acting forces in heavier individuals of the same species require relatively thicker cartilage. But that may only affect larger species than sampled here.

To further investigate the influence of estimated joint spacing (JS) on range of motion (ROM) modelling, we conducted a case study on three rodent species with differing BM and cursorial adaptations. Our results indicate that modeled JS significantly impacts in silico ROM outcomes, particularly in quantitative volumetric analyses of the joint's pose space (mobility). However, relatively, mobility was unaffected by JS, as it consistently decreased from rats to guinea pigs to maras. This was independent of the method used to estimate JS. Overall, mobility comparisons appear to be more robust for assessing differences between species with varying locomotor adaptations and BM than analyses based solely on individual degrees of freedom (DOFs). Only inclusion of all six DOF (three rotational, three translational) resulted consistently in biologically realistic results. While our ROM results cannot distinguish whether observed differences arise from locomotor adaptations or BM, future research should aim to disentangle these effects and expand the scope of quantitative ROM comparisons.

## AUTHOR CONTRIBUTIONS

Adrian Scheidt: concept and design, acquisition of data, data analysis/interpretation, visualization, methodology, drafting of the manuscript, critical revision of the manuscript. Alina CE Renk: acquisition of data, data analysis/interpretation, visualization, methodology, critical revision of the manuscript. John A Nyakatura: concept and design, data analysis/interpretation, revision of the manuscript.

## Supporting information


Data S1:

Data S2:

## Data Availability

The data that support the findings of this study are available in the supplementary material of this article.
