# Supplementary material for: Scaling of internal joint distance in the elbow of small‐ to medium‐sized mammals: Implications for range of motion analyses
Source: J Anat. 2026 Feb 6;248(6):950–71. doi: 10.1111/joa.70116 (PMC13148638; doi:10.1111/joa.70116)
Supplement: Supplementary file 2 — Data S2: [file JOA-248-950-s001.docx]

**Supplementary material**


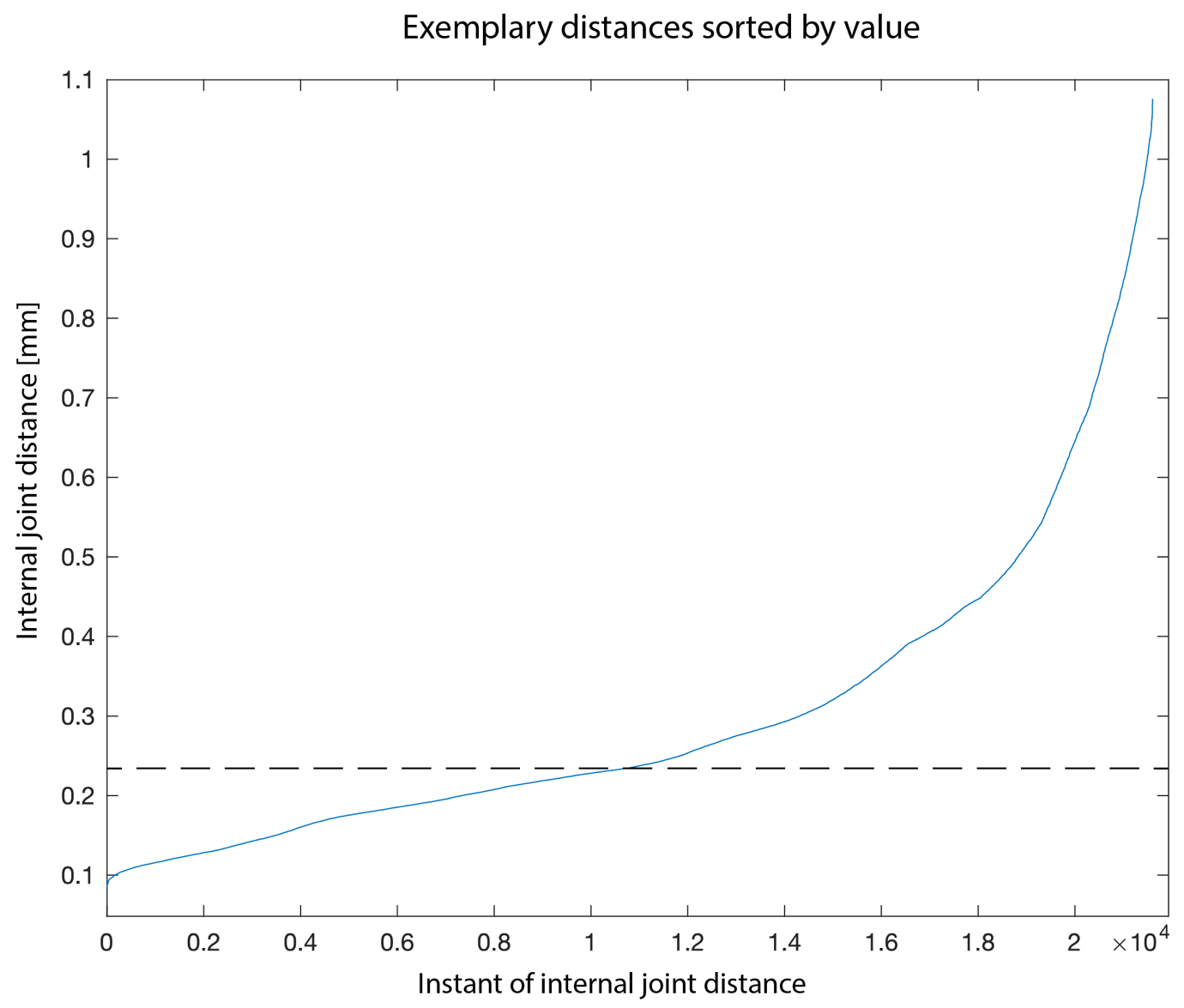


*Figure S1. Exemplary results of internal joint distances in a humero-ulnar articulation of a guinea pig.* The internal joint distance was measured at roughly 21,000 (2.1*10^4^) positions across the ulnar articulation site. In this example, the median value of internal joint distance is 0.2499 mm (dashed line).


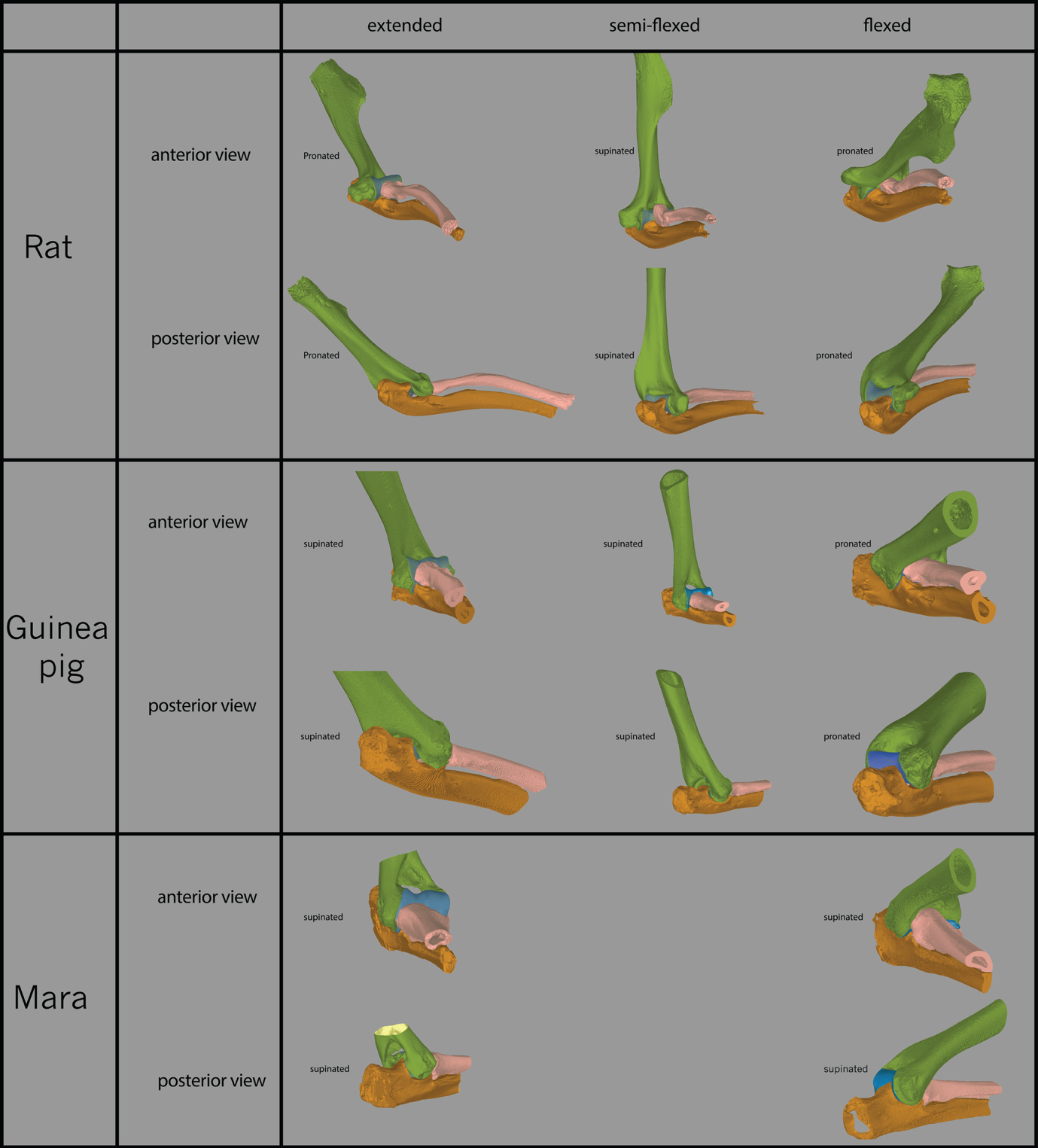


*Figure S2. Details of elbow joints of the pose spectrum analysis.* The medial side of left elbows are shown in extended, semi-flexed, and fully flexed positions for each species. Every pose is displayed in medio-anterior (top) and medio-posterior view (bottom). Meshes of humeri are display in green, ulnae in brown, radii in pink, and humeral articulation sites in blue. Note, that for the mara no semi-flexed position is available (see main text for details). Supination and pronation of the displayed specimens is indicated next to the images. Further, to increase resolution during μCT-scanning and improve computational time during mesh segmentation in Dragonfly, the proximal end of the humerus and the distal ends of radius and ulna are sometimes cut-off. Additionally, the mesh of mara olecranon displays holes which do not occur naturally.


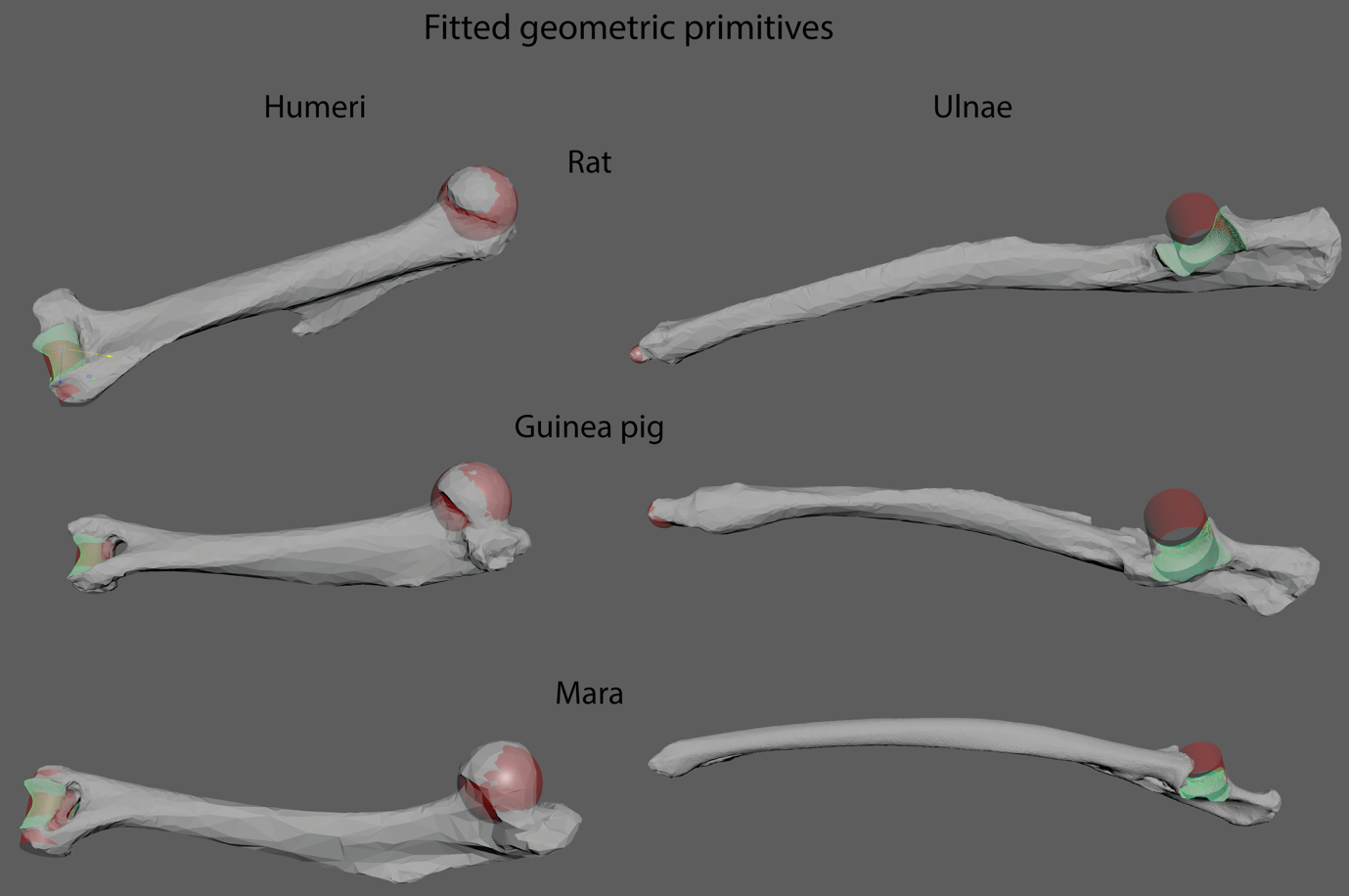
 *Figure S3. Images of bones with fitted geometric primitives.* Right humeri and ulnae of the studied species are depicted. Humeri are displayed in latero-posterior view, ulnae in latero-anterior view. Note that the radius and ulna are fused in maras, thus both bones are displayed here with the radius covering the ulna. The primitives are shown in opaque red, with cylinders fit to the humeral trochlea and the ulnar trochlear notch. The longitudinal axes of the cylinders point roughly in medio-lateral direction. Spheres are fit in the humeral heads and the ulnar styloid processes. The articulation surfaces of the humeral trochlea and ulnar notches are shown in green. Fitting of primitives was done in Geomagic wrap (Version 2017.0.0, 3D Systems Inc., Rock Hill, SC, USA) (see main text for details).


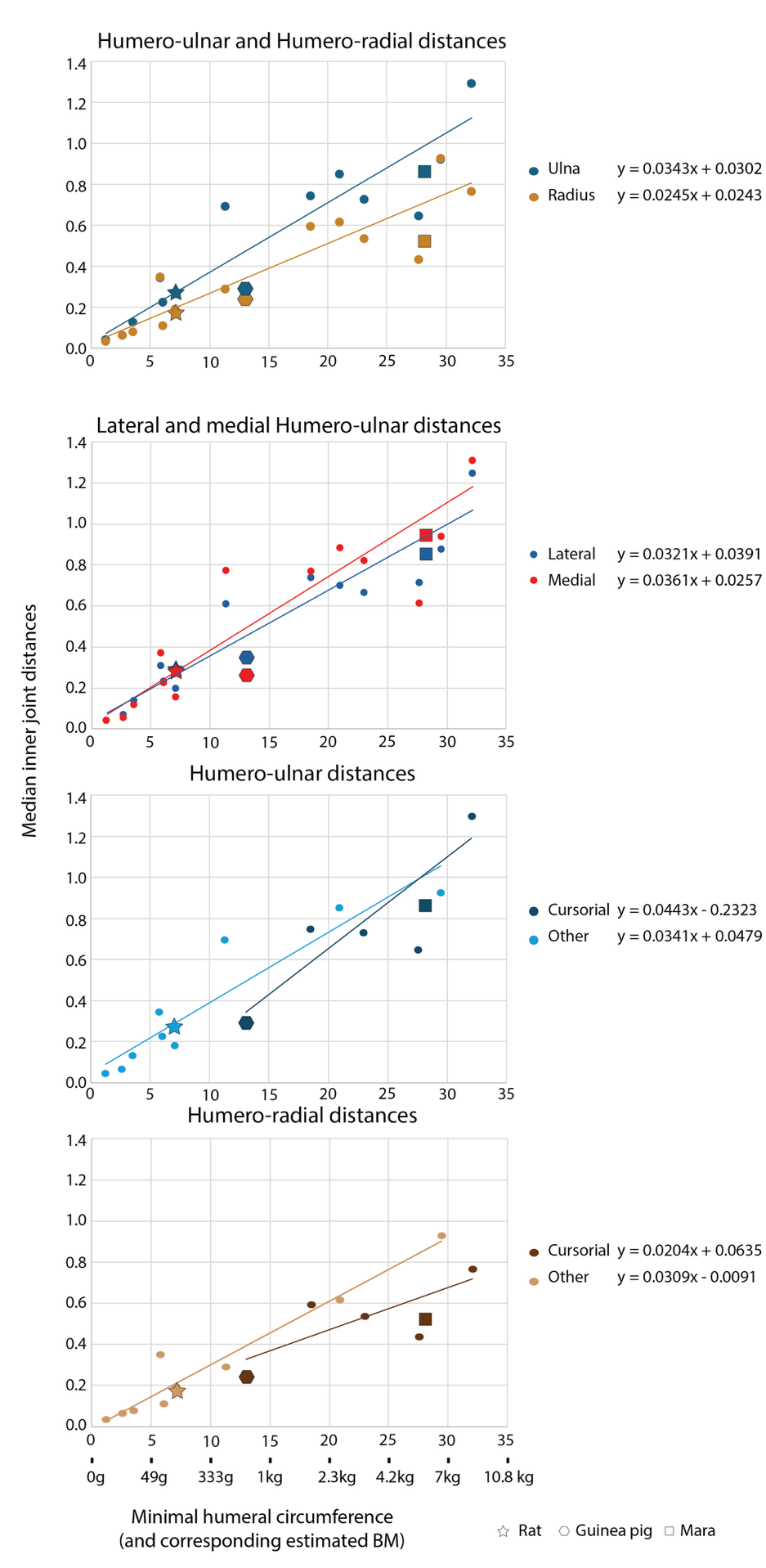


*Figure S4. Non-log transformed results of the* scaling analysis*.* The x- axes shows the minimal humeral circumferences (in mm) with estimated BMs based on Campione and Evans (2012). Y-axes show the median internal joint distances. Star, pentagon, and square symbols represent rat, guinea pig, and mara results respectively. The data here has not been log-transformed, unlike Figure 3 of the main text.


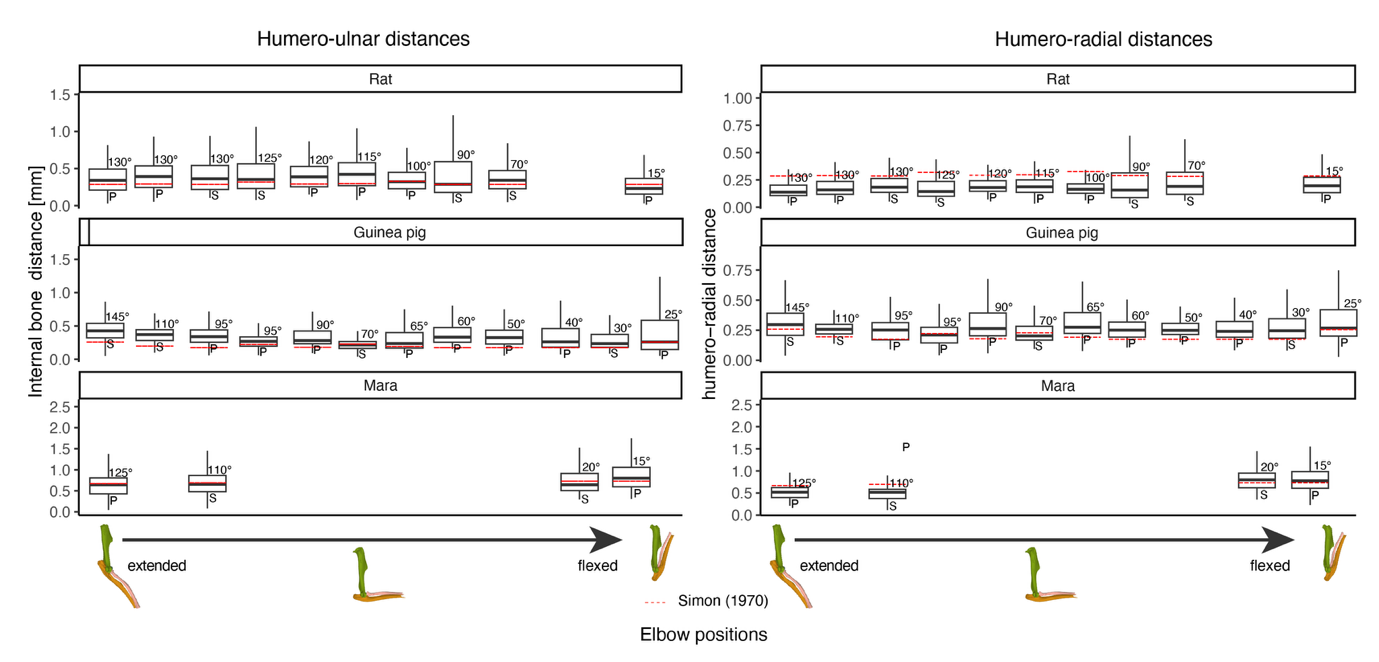


*Figure S5. Non-normalized boxplot of internal joint distances in a pose spectrum*. This graph shows absolute internal joint distances, whereas the data in Figure 4 of the main text has been normalized to specimens’ size (see text for details). Each boxplot represents one elbow of one specimen, sorted by increasing flexion angle. Outliers are not depicted in the graph. Red stripes show the predicted average internal joint distance based on cartilage thickness measures by Simon (1970). Bottom images display the range of extended poses from 130° to 15° in rats, with the humerus in green, ulna in brown, and radius in pink. On top of each boxplot is the degree of extension indicated. P and S refer to pronated and supinated postures, respectively. Note the different scaling of the Y-axes between the results of each species to account for the differences between species in absolute internal joint distances.


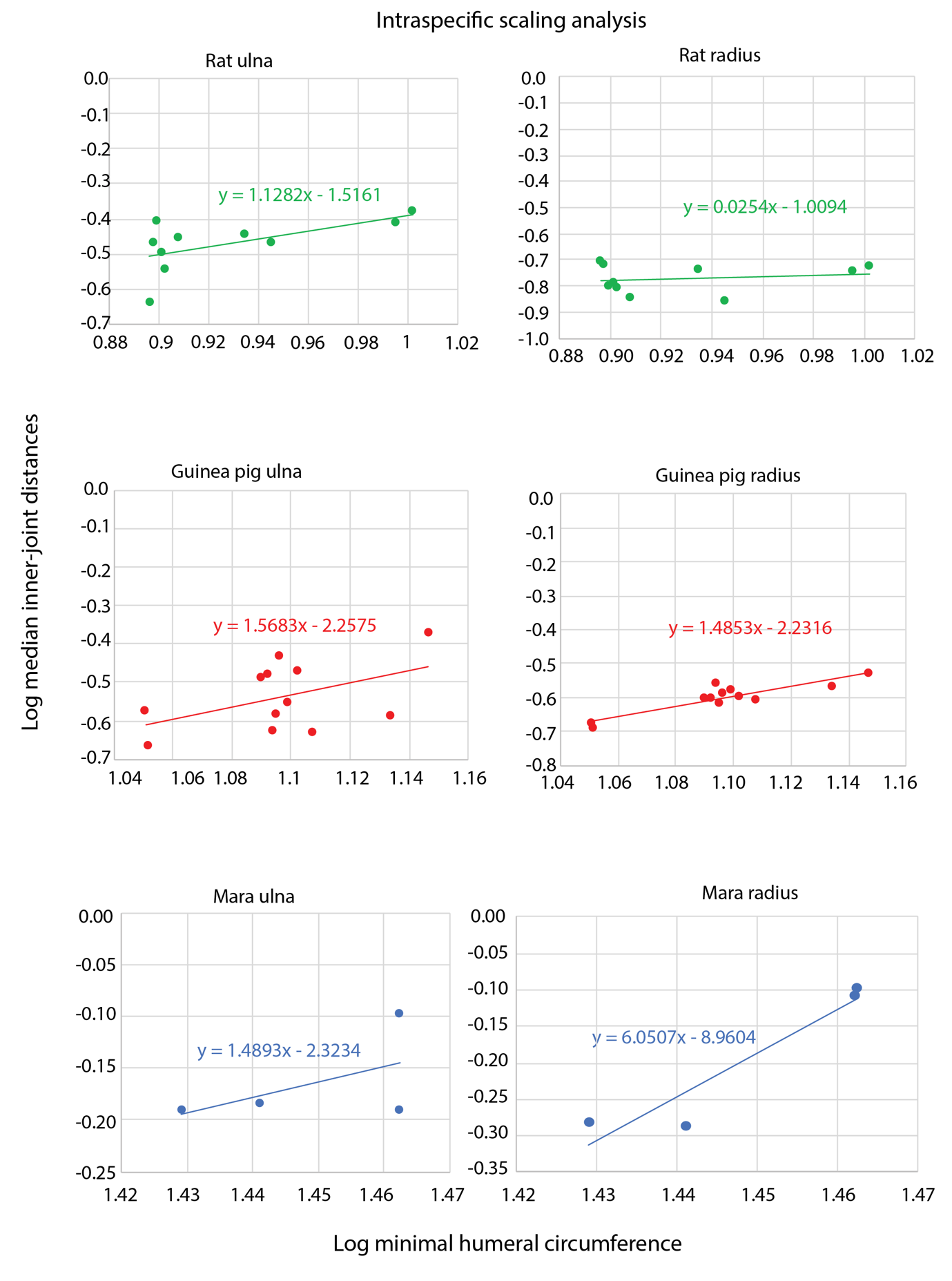


*Figure S6. Intraspecific Scaling analysis.* Log transformed medians of the Humero-ulnar joint on the left, Humero-radial joints on the right. Slopes with b < 1 indicate negative allometry, b = 1 isometry, and b > 1 positive allometry. Statistically, none of the slopes deviate from isometry, as the 95% confidence intervals include values of b = 1 (see main text for details).


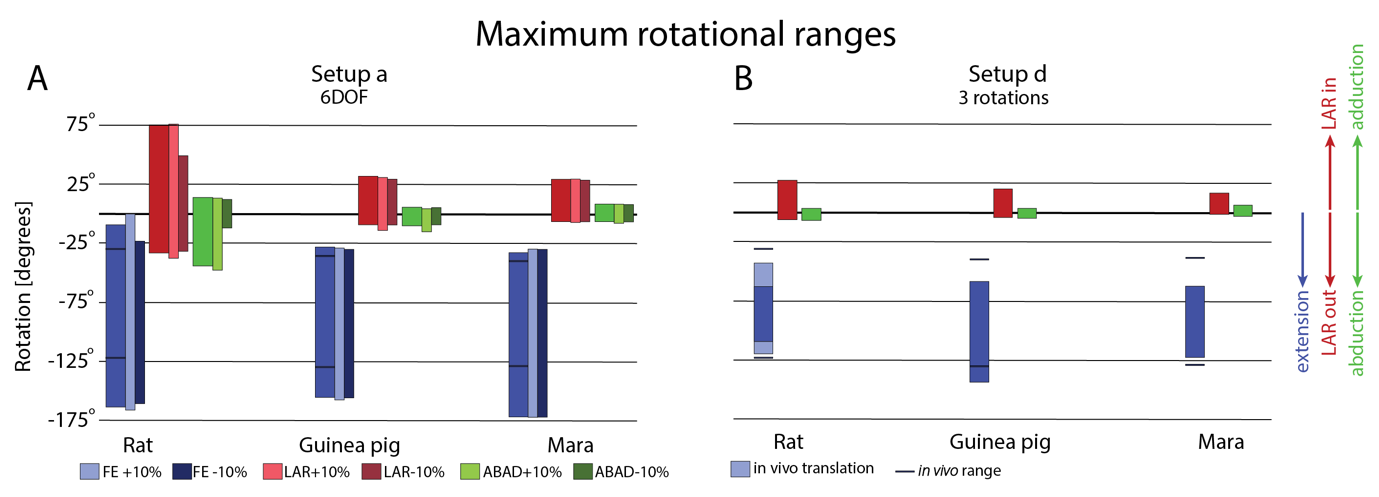


*Figure S7 Maximum ranges of rotational range of motion.* Blue bars represent Flexion/extension (FE), red long axis rotation (LAR) , and green abduction/adduction (ABAD). In (**A**), lighter and darker bars refer to increased or decreased distance threshold in setup a+ and a-, which are not depicted in Figure 5 of the main text. Small horizontal stripes refer to estimated *in vivo* FE ranges of each species (see discussion for details). **B** Setup d, only rotations were allowed freely (3DOF). The light blue bars in rat show the resulting rotational values, when limited translation was allowed based on *in vivo* data by Bonnan et al. (2016), see main text for details. Note, that in this graph *in vivo* ranges are indicated with small bars, opposed to figure 5 in the main text.

*Table S1. Measured internal joint distances in mouse, rat, and dog, and the compared cartilage thickness in the femoral condyles.* Columns marked with an asterisk (*) show values from Malda et al (2013) who measured cartilage thickness in medial and lateral femoral condyles. Columns marked with a plus (+) show data from this study. Med. Femur Cart and Lat Femur Cart. refer to medial and lateral femoral cartilage thickness respectively. The median IJDs of this study were halved, to approximate the bones’ cartilage thickness in the last two columns (see text for details). BM^+^ refers to estimated body mass by minimal humeral circumference (see text for details). This studies’ results of the rats (n= 10) were taken from the *pose spectrum analysis* of this study, and refer to 10 limbs analysed from 5 specimens. The mouse and dog values are incorporated in the *interspecific* *scaling analysis* section and refer to a single limb from 1 or 3 specimens respectively. Note that, while the halved ulnar distances are smaller than the reported cartilage thickness, the specimens here were also of lower body mass. One dog examined with BM = 11.3kg showed halved ulnar distances of 0.843 mm, very similar to the dogs (12kg) from Malda et al (2013) medial femur cartilage thickness.

| **Species** | **BM^*^** | **BM^+^** | **Med. Femur Cart. ^*^**  **[mm]** | **Lat Femur* Cart. ^*^**  **[mm]** | **Radial distance /2^+^**  **[mm]** | **Ulnar distance /2^+^**  **[mm]** |
| --- | --- | --- | --- | --- | --- | --- |
| Mouse | 25g | 18g | 0.087±0.013 | 0.099 ±0.032 | 0.04 | 0.065 |
| Rat | 300g | 217±63g (n=10) | 0.235±0.046 | 0.213±0.029 | 0.085±0.01 | 0.172±0.028 |
| Dog | 12 kg | 10.1±7.5kg  (n=3) | 0.849±0.185 | 0.476±0.146 | 0.383±0.15 | 0.648±0.237 |

Table S2. Results of the test for a phylogenetic signal within the residues, using Blombergs K (K), assuming Brownian motion. A value of K < 1 indicates that the trait has a low phylogenetic signal. The null hypothesis states no phylogenetic signal, thus the trait is randomly distributed across the phylogenetic tree. The null hypothesis was not rejected for any trait.

| Trait | K | p- values |
| --- | --- | --- |
| Radio-humeral distances | 0.394892 | 0.526 |
| Ulnar-humeral distances | 0.462843 | 0.332 |
| Lateral ulnar distances | 0.364024 | 0.594 |
| Medial ulnar distances | 0.482585 | 0.288 |

*
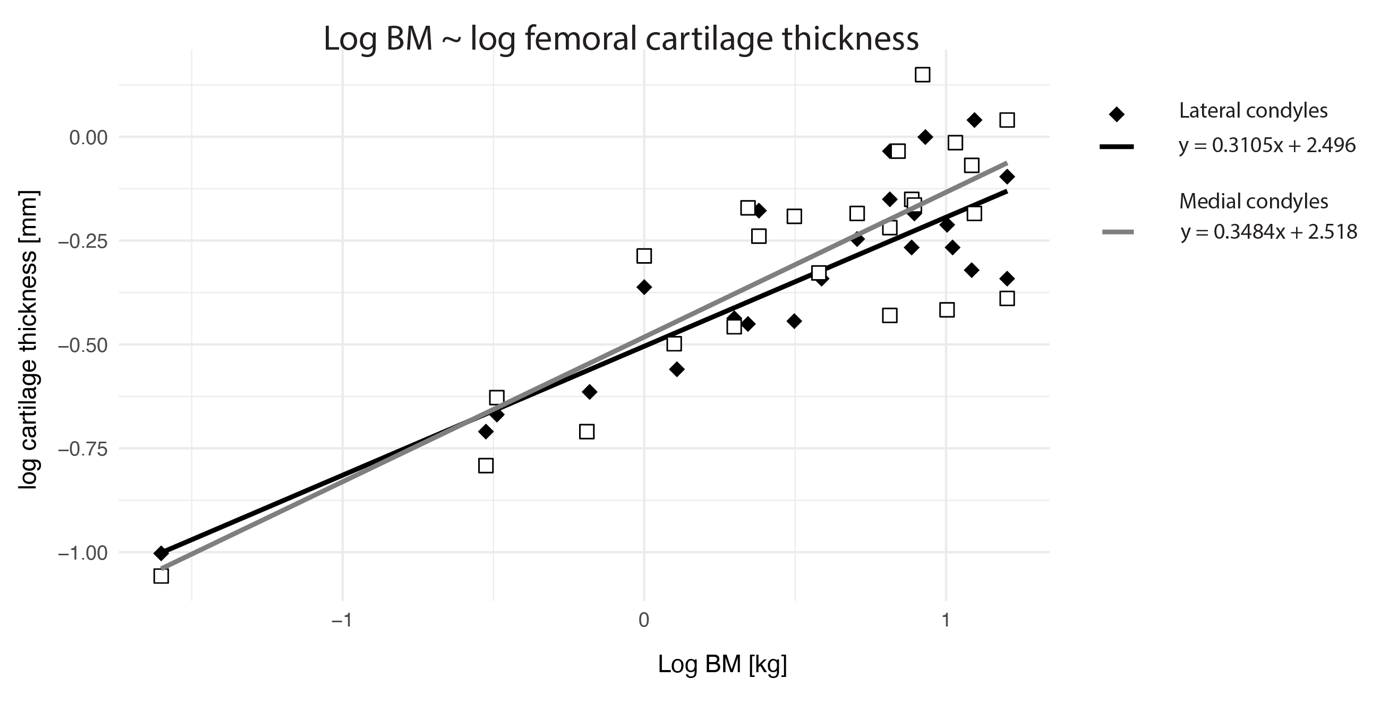
*

*Figure S8. Scaling femoral cartilage thickness in a subsample from Malda et al. (2013).* Data points were retrieved from Figure 3 in Malda et al. (2013) using the webplotdigitizer, Version 5.2, by Ankit Rohatgi (https://automeris.io/wpd/). Data points present the average cartilage thickness in lateral (black diamonds) and medial (white squares) femoral condyles. A slope of 0.3 correspond to isometry, which neither slope deviates from statistically. Malda et al.’s (2013) original data set comprised species up to 4000kg, this graph contains specimens ranging from 0.025 kg (Mouse) to 16kg (Indian crested porcupine).

File S1. This compressed file contains three files containing matlab figures with the results of rat, guinea pig, and mara of the ROM analysis, setup a. The left graph (in blue) shows the alpha shape around cosine-corrected rotational values of the viable poses in a deg-deg-deg space. Each Axes corresponds to one rotational degree of freedom (X-LAR, Y- ABAD, Z- FE). In this example, the alpha shapes were fitted with a critical r alpha = 10. The middle graph (in red) shows the alpha shape around the translational values of the viable poses. Each axis corresponds to one translational degree of freedom (see text for details). Here, alpha shapes were fitted with r alpha = 1. The graphs on the right track the mobility (volume of alpha shape) over generations. One generation is a computational cycle in ‘APSE’ (see Bishop et al., 2023, for details).
